# Replication Vesicles are Load- and Choke-Points in the Hepatitis C Virus Lifecycle

**DOI:** 10.1371/journal.ppat.1003561

**Published:** 2013-08-22

**Authors:** Marco Binder, Nurgazy Sulaimanov, Diana Clausznitzer, Manuel Schulze, Christian M. Hüber, Simon M. Lenz, Johannes P. Schlöder, Martin Trippler, Ralf Bartenschlager, Volker Lohmann, Lars Kaderali

**Affiliations:** 1 Heidelberg University, Medical Faculty, Department of Infectious Diseases, Molecular Virology, Heidelberg, Germany; 2 Technische Universität Dresden, Institute for Medical Informatics and Biometry, Dresden, Germany; 3 Heidelberg University, ViroQuant Research Group Modeling, BioQuant BQ26, Heidelberg, Germany; 4 Heidelberg University, Interdisciplinary Center for Scientific Computing (IWR), Simulation and Optimization Group, Heidelberg, Germany; 5 University Hospital of Essen, Department of Gastroenterology and Hepatology, Essen, Germany; University of Texas at Austin, United States of America

## Abstract

Hepatitis C virus (HCV) infection develops into chronicity in 80% of all patients, characterized by persistent low-level replication. To understand how the virus establishes its tightly controlled intracellular RNA replication cycle, we developed the first detailed mathematical model of the initial dynamic phase of the intracellular HCV RNA replication. We therefore quantitatively measured viral RNA and protein translation upon synchronous delivery of viral genomes to host cells, and thoroughly validated the model using additional, independent experiments. Model analysis was used to predict the efficacy of different classes of inhibitors and identified sensitive substeps of replication that could be targeted by current and future therapeutics. A protective replication compartment proved to be essential for sustained RNA replication, balancing translation versus replication and thus effectively limiting RNA amplification. The model predicts that host factors involved in the formation of this compartment determine cellular permissiveness to HCV replication. In gene expression profiling, we identified several key processes potentially determining cellular HCV replication efficiency.

## Author Summary

Hepatitis C is a severe disease and a prime cause for liver transplantation. Up to 3% of the world's population are chronically infected with its causative agent, the Hepatitis C virus (HCV). This capacity to establish long (decades) lasting persistent infection sets HCV apart from other plus-strand RNA viruses typically causing acute, self-limiting infections. A prerequisite for its capacity to persist is HCV's complex and tightly regulated intracellular replication strategy. In this study, we therefore wanted to develop a comprehensive understanding of the molecular processes governing HCV RNA replication in order to pinpoint the most vulnerable substeps in the viral life cycle. For that purpose, we used a combination of biological experiments and mathematical modeling. Using the model to study HCV's replication strategy, we recognized diverse but crucial roles for the membraneous replication compartment of HCV in regulating RNA amplification. We further predict the existence of an essential limiting host factor (or function) required for establishing active RNA replication and thereby determining cellular permissiveness for HCV. Our model also proved valuable to understand and predict the effects of pharmacological inhibitors of HCV and might be a solid basis for the development of similar models for other plus-strand RNA viruses.

## Introduction

Hepatitis C virus (HCV) infection is a major global health problem, with approximately 170 million chronically infected individuals worldwide and 3 to 4 million new infections occurring each year [1]. Acute infection is mostly asymptomatic, however, it develops into a chronic infection in about 80% of patients, and then is a leading cause of liver cirrhosis, hepatocellular carcinoma and subsequent liver transplantation [2], [3], [4]. A significant fraction of patients cannot be cured even with modern combination therapies, partially due to *ab initio* non-responsiveness, but also due to the emergence of drug-resistant HCV quasispecies.

HCV is an enveloped plus-strand RNA virus and belongs to the *Flaviviridae* family. Upon entry into the host cell, its 9.6 kb genome is translated by a cap-independent, internal ribosomal entry site (IRES) mediated mechanism into a single large polyprotein. Viral and cellular proteases co- and post-translationally cleave this precursor into ten mature viral proteins, comprising three structural proteins (core, E1, E2), the ion channel p7 as well as the six non-structural (NS) proteins NS2, 3, 4A, 4B, 5A and 5B [5]. The five “replicase” proteins NS3 to NS5B are essential and sufficient for intracellular genome replication. NS3 comprises an RNA helicase and a protease domain, the latter of which, together with the co-factor NS4A, forms the major viral protease NS3/4A, liberating itself and all other replicase proteins from the polyprotein precursor. NS4B, together with other NS proteins, induces membrane alterations, observable as convoluted, vesicular membrane structures known as the membranous web and believed to act as the sites of RNA replication [6], [7]. The exact architecture and topology of these structures, and particularly their structure-function-relationship, is not fully understood yet. However, for Dengue virus (DV), a related flavivirus, the three-dimensional makeup of the membrane rearrangements has been solved recently [8]. There, numerous small, vesicular invaginations into the rough endoplasmic reticulum (ER) serve as a protected environment for genome replication. NS5A is a phosphoprotein important both in RNA replication and particle assembly and/or release. NS5B, the RNA-dependent RNA polymerase (RdRP), is the core enzyme of the replicase complex. In order to amplify the viral RNA, NS5B first synthesizes a complementary (i.e. negatively oriented) strand from the plus stranded genome, putatively resulting in a double-stranded (ds) intermediate [9]. From this negative strand template, NS5B then transcribes progeny plus strands. Given the ∼10-fold higher number of plus strands over minus strands within the host cell, this most likely occurs in a repetitive manner [10]. Newly synthesized plus strands are then released by an unknown mechanism from the replicative compartment and can then either be directed to encapsidation into assembling virions, or re-enter the replicative cycle by serving as templates for further translation and subsequent incorporation into a new replication complex.

It is interesting to note that although HCV establishes a persistent infection, it does not have a latent phase; throughout the course of the infection, which can be decades long in many patients, there is constant production of viral RNA, proteins and infectious particles. In most viral infections, presence of non-self structures, such as dsRNA or viral proteins, is readily detected by sensors of the immune system, leading to the production of type I interferon (IFN) and activation of the adaptive immune response [11]. Also in case of HCV, innate as well as adaptive immune responses are elicited, however, by means of various complex interactions with cellular processes, the virus is capable to blunt these defense mechanisms and thus is able to persist [12]. This ability of HCV to maintain low profile persistence is most likely intimately linked to its tightly controlled RNA replication; for the closely related bovine diarrhea virus (BVDV), which can be converted from a persistently to an acutely replicating form, a direct correlation between excessive RNA replication and the induction of cytopathic effects has been described [13]. To comprehensively study these complex and highly dynamic processes that can only inappropriately be addressed by individual experiments, an eminent approach is mathematical modeling. Consequently, a basic model of HCV infection dynamics was published almost 15 years ago [14] and has since led to the development of several related models of HCV infection and therapy dynamics [15], [16], [17], [18], [19], [20], [21]. However, all of these models described the long-term dynamics at the level of cell populations, organs and even organisms (patients), and did not take intracellular processes such as genome translation and the actual RNA replication into account. With the development of subgenomic HCV replicons, detailed studies of intracellular RNA replication became possible [22], [23]. A thorough quantitative analysis of persistent subgenomic replicons in Huh-7 cells [10] led to the development of a first mathematical model of intracellular steady state RNA replication [24]. Further models addressed the effect of potential drugs on viral replication [25] or included the production of virus particles [26], [27]. However, all published models were solely based on measurements of steady state replication. In contrast, to understand how the virus on the one hand manages to efficiently (and quickly) establish itself within a host cell before the cell is able to mount an antiviral response, while on the other hand, it is strictly limiting its own amplification, static (steady state) data is not sufficient but needs to be complemented by information about the dynamic aspects of replication. Previous studies on replication kinetics in cell culture in fact point to a highly dynamic initial phase of RNA replication in the first few hours after genome transfection or infection, which then reaches a steady state within 24–72 hours [22], [28], [29]. Actual amplification kinetics and the absolute levels attained in the steady state vary largely between different experimental systems and are mainly determined by the permissiveness of the employed host cell [30], [31], [32] and by the viral isolate [30], [32], [33].

Therefore, in our present study we quantitatively followed the onset of intracellular RNA replication within the first couple of hours upon introduction of HCV genomes into the host cells. Based on these data we developed a comprehensive mathematical model capable of precisely describing both, the dynamic and the steady state phases of viral replication. We then used this model to study aspects of the viral replication cycle that cannot directly be accessed experimentally.

## Results

**Figure 1:**
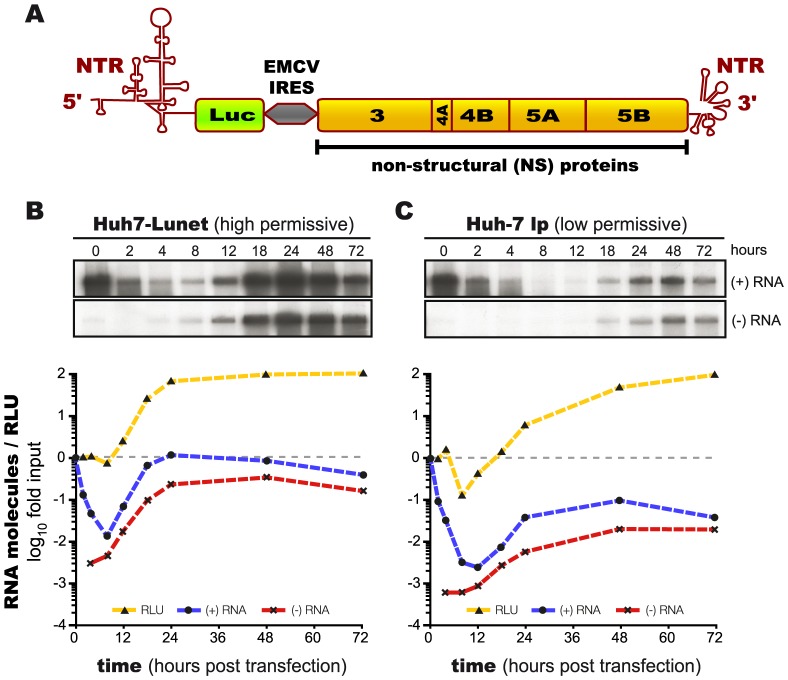
Measurement of HCV replication dynamics. (A) Schematic representation of the subgenomic HCV luciferase reporter replicon used for the study. The 5′-non-translated region (NTR) contains the HCV internal ribosome entry site (IRES), controlling translation of the firefly luciferase gene (Luc). The non-structural proteins of the HCV isolate JFH1 are under control of the encephalomyocarditis (EMCV) virus IRES, and are followed by the orthologous 3′- NTR of JFH1. (B&C) Quantitative assessment of the HCV replication dynamics upon instantaneous (t = 0 h) electro-transfection into (B) high permissive Huh7-Lunet cells or (C) low permissive Huh-7 low passage cells. The top panel shows a Northern blot analysis of the viral plus- and minus-strand RNA. The lower panel shows a graph of the Northern blot signals quantified by phosphor imaging (plus-strand RNA: blue lines; minus-strand RNA: red lines), as well as the corresponding luciferase activity (RLU, yellow lines). Luciferase activity and plus-strand RNA are normalized to the input values (2 h and 0 h, respectively; one representative experiment is shown. Lines in the plots are for illustrative purposes and connect data points, but are not results of mathematical modeling.

To assess the dynamics of HCV RNA replication, we performed quantitative, time resolved measurements of strand specific viral RNA and polyprotein concentrations over 72 h after viral RNA transfection into Huh7 cells. To achieve sufficiently strong replication that can be measured reliably, we used subgenomic reporter replicons carrying the firefly luciferase gene in front of the viral proteins [28] (figure 1A), and we synchronized the onset of replication to the largest feasible extent by using electroporation to instantaneously introduce *in vitro* transcribed replicon RNA into the cells. As host cellular factors play a critical role in determining the efficiency of viral replication [30], [31], we used two different cell lines: Huh7-Lunet is a clonal cell line of exceptionally high permissiveness for HCV RNA replication [34], whereas a low passage of standard Huh-7 cells (Huh-7 lp) replicates HCV RNA to significantly lower levels, as has been described previously [30]. Over the course of 72 hours we then followed HCV replication, measuring plus-strand and minus-strand RNA by strand specific quantitative Northern blotting and firefly luciferase activity as a highly sensitive surrogate marker of viral protein translation, since luciferase expression was under the control of the HCV IRES (see figure 1A). Of note, luciferase activity correlates with the amount of viral protein translated, but does not allow discrimination between cytoplasmic NS proteins and proteins inside the RC.

Upon transfection of replicon RNA into Huh7-Lunet cells, the RNA was instantly translated into protein and at the same time was rapidly degraded (figure 1B). Consequently, after a first peak, translation also leveled off or even decreased slightly, while negative strand RNA first became detectable at 4–8 hours post transfection. From around 8 hours on, synthesis of new positive strand RNA then exceeded its degradation, and levels of both, positive and negative strand RNA as well as of viral protein started to increase rapidly (note the logarithmic scale in figure 1B). A steady state was finally reached at around 30 hours post transfection (in Huh7-Lunet), which was stable until the end of the observation at 72 hours.

### Establishing of a base model to describe initial HCV RNA replication dynamics

**Figure 2:**
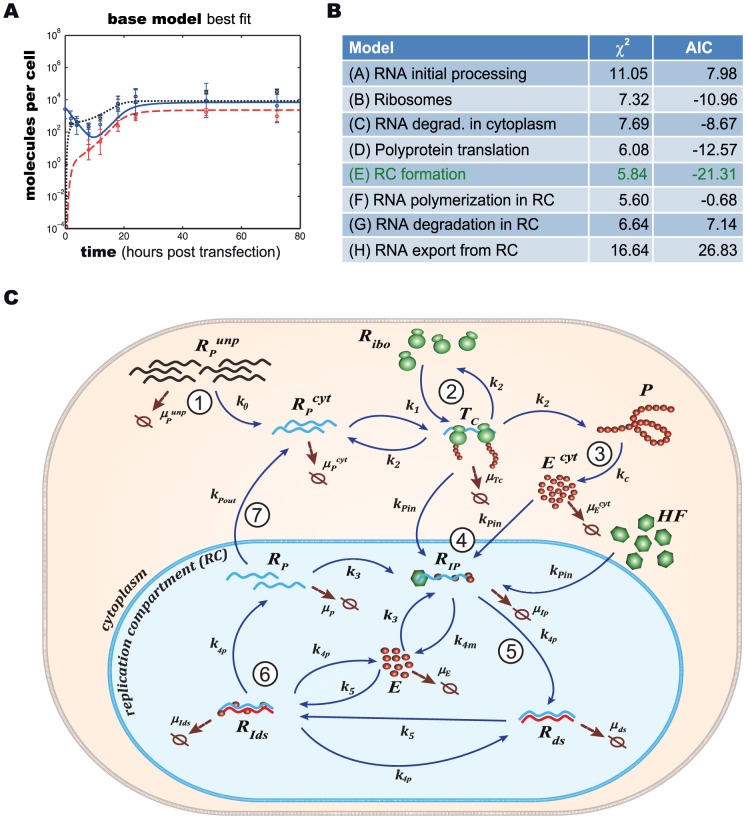
Model development and model selection. (A) Model simulation of our calibrated base model (comprising the model by Dahari et al. [24], with an added initial processing step for transfected RNA and cis-triggered formation of the replication compartment,) compared to experimental data for high-permissive Huh 7-Lunet cells. Black: Polyprotein, red: plus-strand RNA, blue: minus-strand RNA. (B) Different hypotheses for the involvement of a host function at all feasible steps in the viral lifecycle were assessed to explain differences observed in the replication dynamics of Huh7-Lunet and Huh-7 lp cells. For each hypotheses, the base model was calibrated simultaneously to data from high- and low-permissive cell lines, allowing only parameters to differ between the two cell lines that are involved in the respective process. The table shows resulting residual squared errors (*χ* ^2^) and computed values of the Akaike Information Criterion, a measure that balances goodness-of-fit with the degrees of freedom of a model. Time courses for the individual fits are shown in supplementary figure S1. (C) Graphical illustration of the final model. The main steps are: (1) viral RNA enters the cell, e.g. via transfection (in our subgenomic replicon experiments) or via receptor mediated endocytosis (in a natural infection setting). RNA then undergoes some structural preprocessing (eq. 1), leading to an increased stability and availability to the translation machinery (as *R_p_^cyt^*, eq. 2). (2) Ribosomes bind the viral RNA, forming translation complexes (*T_c_*, eq. 3) and translate it into a polyprotein (*P*, eq. 4); (3) the polyprotein is subsequently cleaved into the mature viral proteins (*E^cyt^*) with rate *k_c_* (eq. 5); (4) viral proteins then induce the formation of a membranous replication compartment (RC), into which actively translated plus-strand RNA (*T_c_*), viral NS proteins (*E_cyt_*) and one or more host factors (subsumed as *HF*) enter with rate *k_Pin_*, forming the plus-strand replication initiation complex (*R_ip_*, eq. 7); (5) complementary minus-strand is then transcribed with rate *k_4m_*, and the complex dissociates into dsRNA (*R_ds_*, eq. 8) and viral polymerase (*E*, eq. 9); (6) dsRNA and polymerase can then re-associate (*R_Ids_*, eq. 10) with rate *k_5_* and synthesize progeny plus-strand genomes (*R_p_*) at rate *k_4p_* (eq. 11); (7) eventually, new positive strand RNA (*R_p_*) is liberated from the replication vesicles into the cytoplasm at rate *k_pout_* (eq. 11 and 2) or, alternatively, can remain within the vesicles for further genome replication (initiating at rate *k_3_*), and is ultimately degraded.

In order to comprehensively understand the observed HCV replication dynamics and its underlying molecular processes, we set up a mathematical model of the intracellular HCV RNA replication. Dahari and colleagues developed a similar model previously, upon which we could build here [24]. Briefly, our model comprises all relevant molecular species (RNA, proteins, ribosomes, etc.), and describes each step in the RNA replication cycle, such as translation, protein maturation and the formation of the actual RNA replication complex, as reactions of the involved molecules using differential equations based on standard mass action kinetics. Of note, the establishment of a vesicular replication compartment (RC) by viral proteins (in concert with cellular functions) within which RNA replication takes place is reflected in the model by partitioning of the respective molecular species into distinct “cytoplasmic” and “replication compartment” pools; e.g. only cytoplasmic HCV RNA (*R_P_^cyt^*) can be translated by ribosomes, but not HCV RNA within the replication compartment (*R_P_*). Model equations (eq.) of our final model are given in the materials and methods section and a schematic illustration is shown in figure 2C. The original model of Dahari was solely based on steady state measurements of viral RNA and protein concentrations in a stable replicon cell line [10], and accordingly was not capable of explaining the dynamic phase during the establishing of replication as observed in our experimental data, even after re-fitting all model parameters (high permissive cell line; total sum of squared residuals *χ* ^2^ = 8.69, compare supplementary figure S1). From this finding it was evident, that modifications to the model were required in order to accurately capture the initial dynamics of HCV RNA replication, as it can be observed upon transfection of viral genomes into “naïve” cells.

Based on biological reasoning, we extended and modified Dahari's original model at two steps of the replication cycle. For one, to account for *ab initio* replication in our setting (in contrast to pre-formed steady-state replication), we introduced one additional RNA species *R_p_^unp^*, representing the transfected “naked” replicon RNA and an according processing step (rate k_0_), subsuming any re-folding, association with RNA-binding proteins and other processes that might take place and be required before *in vitro* transcribed RNA assumes a translation-competent state (eq. 1 and 2). This processing corresponds to viral genomic RNA being released into the cytoplasm upon actual infection. We furthermore allowed RNA degradation to be different (presumably higher) for the “unprocessed” transfected RNA (*μ_p_^Unp^*) as compared to “processed” or cell-derived RNA (*μ_p_^cyt^*). The second step that we updated to reflect the current biological understanding of the molecular processes was the initiation of minus strand RNA synthesis (which in the model is assumed to correspond to the formation of the replicative compartment, as discussed later). It has been described for HCV, but also for other viruses [35], [36], [37], [38], that the formation of a productive replicase complex requires the viral polymerase (NS5B) and possibly other NS-proteins to be supplied in *cis*. This means that for reasons not yet fully elucidated, NS5B cannot initiate RNA synthesis from a free, cytosolic RNA genome, but only from the very RNA that it has been translated from. This implies a tight spatio-temporal coupling of (poly)protein production and initiation of RNA replication, i.e. initiation can only occur immediately after translation/polyprotein processing and therefore in close proximity to the translation complex (*T_C_*). As our model does not account for spatial effects (such as diffusion), we approximated this *cis*-process by requiring an active translation complex instead of free, non-translating RNA (*R_P_^cyt^*) for the initiation of minus strand RNA synthesis (*R_IP_*, eq. 7). This *cis*-triggered formation of the replicative compartment consequently is the only route for uptake of viral genomes and also NS proteins (*E^cyt^*) into replication vesicles. This is a major change to Dahari's original model, in which cytosolic RNA (*R_p_^cyt^*) and NS proteins (*E^cyt^*) could freely and independently enter the compartment. This model, comprising Dahari's original model with the described extensions, we then considered our base model.

We then tested, whether our base model would be capable of explaining the measured replication dynamics. We therefore fitted the model to the experimental data from the high permissive Huh7-Lunet cells. In fact, this resulted in a significantly better fit as compared to the original model (Dahari: *χ* ^2^ = 8.69, base model: *χ* ^2^ = 2.12) and was capable of adequately describing both, the highly dynamic initial phase as well as the ensuing steady state of viral RNA replication (figure 2A).

### Host factor involved in formation of replication vesicles is sufficient to explain replication dynamics in differently permissive cells

Having established a base model for HCV replication, we next wanted to assess which factors could explain differences observed between high and low permissive cell lines. In our experimental measurements for two differently permissive cell lines, Huh7-Lunet (high permissive) and Huh-7 lp (low permissive), replication reached a steady-state within the period of observation (72 h), however, plateau levels of viral protein, plus-strand RNA and minus-strand RNA differed by approximately one order of magnitude; further, the onset of the net increase of plus-strand RNA was delayed significantly in the low permissive cells and also the minimum concentration of plus-strand RNA reached during net degradation in the first hours after transfection were significantly lower in low permissive cells (compare figure 1B and C). As both, Huh7-Lunet and Huh-7 lp cells, were transfected with the same subgenomic HCV replicon, these differences must be due to differences between the host cells. In order to reflect this host influence also in our model, we tested different steps in the HCV RNA replication cycle that do or could feasibly depend on a host process: (A) efficiency of RNA entry or initial RNA processing; (B) the number of ribosomes available for RNA translation; (C) RNA degradation in the cytoplasm (possibly including antiviral processes such as activation of RNaseL); (D) polyprotein translation or maturation (i.e. cleavage); (E) the formation of the replicative compartment/initiation of minus-strand synthesis; (F) RNA synthesis or (G) RNA degradation inside the replication compartment; or (H) the export of newly synthesized RNA into the cytoplasm. To evaluate these alternatives for their capacity to explain the differences in dynamics and steady-state levels between the two cell lines, we fitted our base model simultaneously to the experimental data from both cell lines, leaving only the parameters free to differ between high and low permissive cells that, in the respective hypothesis (A) to (H), depend on the corresponding host factor; all other parameters were constrained to be identical between the two cell lines. We found that hypotheses (A), (B), (C), (D), (F), (G) and (H) could not explain the above described qualitative difference in replication dynamics: while (C) and (H) did lead to a steady-state but could not reproduce the lower plateau RNA levels in Huh-7 lp, hypotheses (A), (B), (F) and (G) altogether failed to establish a steady-state in low permissive cells in the course of the simulated time period of 80 h (supplementary figure S2). In order to identify the best fitting hypothesis, we also quantitatively assessed the capability of each hypothesis to fit both data sets by calculating *χ* ^2^ over all data points from the two time series, as well as Akaike's information criterion (AIC), which additionally takes into account the number of unconstrained parameters (figure 2B). While parameter differences in the RNA synthesis inside the RC, i.e. hypothesis (F), led to the lowest overall *χ* ^2^ value, hypothesis (E)– assuming a difference in the formation of the RC and initiation of RNA synthesis– led to a slightly larger *χ* ^2^ (5.84 vs. 5.60) but a significantly lower AIC (−21.31 vs. −0.68). Moreover, hypothesis (E) reached a steady-state within 80 h, while (F) did not. This comparison therefore identified the initiation of minus strand RNA synthesis (i.e. the formation of the RC) as the step in the model, at which alteration of a single reaction rate suffices to optimally transform replication dynamics from high permissive cells into the dynamics found in low permissive cells.

Biologically, this step is highly complex and not thoroughly understood yet. After translation and polyprotein processing, reorganization of host cell endomembranes is triggered by viral NS proteins such as NS4B, which has been shown to be a key player in the formation of membrane convolutions at the rough endoplasmic reticulum. These vesicular membrane structures, dubbed the membranous web, have been reported to be the site of HCV RNA replication [7], providing a distinct replicative compartment for the viral replicase machinery. However, the molecular mechanisms leading to the formation of productive replication vesicles are not known. Nonetheless, it is clear that host factors must be required in this complex process, for example proteins involved in membrane biogenesis and reorganization, as well as signal transducers and regulatory molecules; and also the initiation of minus strand RNA synthesis might require a cellular co-factor. It appears plausible that limited abundance of one of these factors in some cells might be responsible for their lower permissiveness for HCV replication. Therefore, we next wanted to include this host factor as an explicit species in our model, which is required for RC formation/minus strand initiation. For that purpose, we subsumed all these possible host determinants by one unspecified host factor *HF* (see figure 2C), which we assumed to interact with viral NS proteins (*E^cyt^*, e.g. NS4B or NS5A) and with actively translated HCV RNA (*T_C_*) to create replication vesicles and to allow for initiation of minus-strand RNA synthesis (being part of the minus-strand initiation complex *R_IP_*, see eq. 7 and figure 2C). In addition, we made the (non-crucial, see supplementary figure S3 and supplementary table S5) assumption that *HF* is only catalyzing the reaction without being consumed.

**Figure 3:**
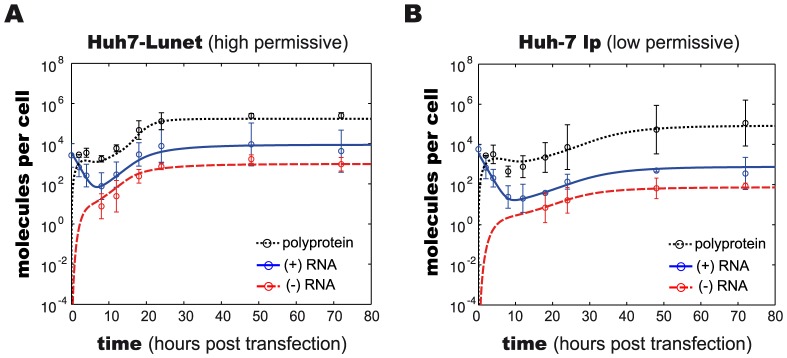
Experimentally measured and model predicted time courses of viral RNA replication. Experimental data (symbols) and results of model simulation (lines) over 80 hours, showing the dynamics of viral replication in (A) high permissive Huh7-Lunet and (B) low permissive Huh-7 lp cells. Solid blue lines and symbols: viral plus-strand RNA; dashed red lines and symbols: viral minus-strand RNA; dotted black lines and symbols: rescaled luciferase activity (i.e. polyprotein molecule numbers). Experimental data represent mean values +/− two standard deviations from three independent replicates. Note the logarithmic scale of the y-axes. Model predictions were obtained after calibration of model parameters to the data.

With this additional modification to the mathematical description of the formation of replication compartments, and calibration of the model to the experimental data from both cell lines (constraining parameters and initial values to biologically meaningful ranges taken from measurements or literature wherever possible), excellent agreement between the model and experimental data was achieved, both, for high and low permissive cells with an overall *χ* ^2^ of 2.01 and AIC of −112.31(figure 3A and B). We therefore considered this our final working model, illustrated in detail in figure 2C. Briefly, the model comprises 13 molecular species in two distinct compartments, the cytoplasm and a replicative compartment (RC), and is parameterized with 16 parameters, corresponding to reaction rates, as well as three non-zero initial values: the initial concentration of HCV RNA (*R_p_^unp^*), the initial concentration of the host factor (*HF*), as well as the total number of ribosomes available for viral RNA translation (*R_ibo_^tot^*). The full system of differential equations and detail on the modeling procedure can be found in the Materials & Methods section; more detail on parameter optimization and analysis are given in supplementary text S1.

Interestingly, analysis of the fitted parameters showed that the concentration of the host factor was more than 10 fold higher in highly permissive Huh7-Lunet cells than in low permissive Huh-7 lp cells. This difference led to slower formation of the replication compartment in Huh-7 lp cells, which in turn resulted in the observed delay in early viral replication and in decreased steady state levels in these cells. Based on our model and computational analysis, we therefore propose that a host process is critically involved in the formation of replication vesicles and/or the initiation of minus-strand RNA synthesis, turning this into the rate-limiting step for HCV RNA replication in low permissive cells.

### Model validation by targeted intervention

While the model could be very well fitted to the original replication data, we then wanted to corroborate its applicability for predicting replication dynamics also under distinct conditions that were not part of the calibration process. For this purpose, we performed additional, independent experiments using mutant HCV replicons with defects at defined stages of the replication cycle. We predicted the impact of such defects on viral replication *a priori* using the model, and retrospectively compared the results with the experimental data in order to assess the validity of model predictions. This approach of introducing targeted mutations into the HCV genome interfering with distinct functions in the viral RNA replication cycle allows validation of individual steps in the model, thus step-wise reconfirming model assumptions and parameters.

**Figure 4:**
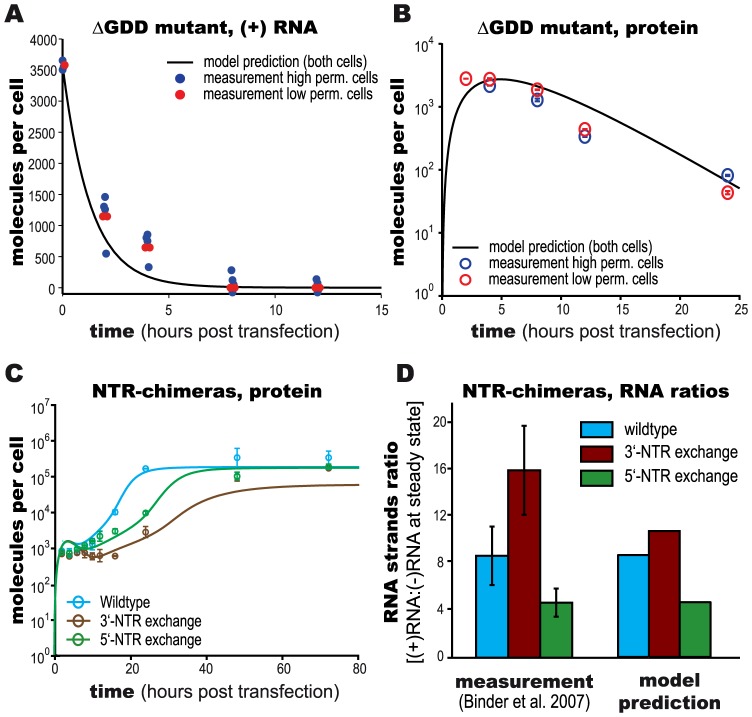
Validation of model predictions. (A+B) Model validation using replication deficient HCV RNA (NS5BΔGDD) in high- and low permissive cells. (A) Plus-strand RNA concentration and (B) protein translation (luciferase activity) were measured. Solid lines indicate model predictions. (C+D) Model validation using chimeric NTR HCV replicons. Exchange of 5′-NTR (green symbols) specifically inhibits initiation of plus-strand synthesis, 3′-NTR exchange (brown symbols) inhibits initiation of minus-strand synthesis. Luciferase measurements are shown as means +/− two standard deviations of two independent experiments. Lines represent model predictions. (D) Comparison of model prediction and literature data [22] for resulting plus- to minus-strand RNA ratios.

As a test of the translation phase of the model, we measured viral plus-strand RNA and protein expression using a replication deficient replicon harboring a deletion of the catalytic triad (GDD motif) of the NS5B polymerase. The measured RNA and protein data thus reflect only the effect of translation and degradation in the cytoplasm, with no RNA replication occurring. We predicted the impact of this intervention with our model by setting the formation rate *k_Pin_* of the plus strand replication initiation complex *R_Ip_* to zero (eq. 3, 5 and 7), thus completely switching off polymerase activity at the earliest possible point, while leaving all other model parameters unchanged. Notably, our model predictions of this intervention matched the experimental data from both, Huh7-Lunet and Huh-7 lp cells, validating our model of cytoplasmic RNA degradation and translation (Figures 4A and B). The fact that the experimental measurements showed almost identical RNA decay dynamics and viral protein (luciferase) levels in high and low permissive cells is also direct experimental confirmation of our modeling based assessment above, that differences in permissiveness cannot be related to RNA “processing” or degradation, or to ribosome availability or protein translation in the cytoplasm (hypotheses (A), (B), (C) and (D) tested above).

We next focused on validating the RNA replication steps of our model. For this purpose, we utilized chimeric replicons with heterologous 5′- or 3′-NTRs derived from a different genotype [22]. We previously showed that these chimeric replicons exhibit decreased replication efficiency due to inefficient initiation of plus-strand synthesis (in case of the 5′-NTR exchange) or minus-strand synthesis (in case of the 3′-NTR exchange) [22]. We predicted the effect of these interventions with the fitted model by decreasing the parameters *k_pin_* and *k_4m_* for the 3′-NTR exchange (eq. 3, 5, 7, 8 and 9), and *k_5_* and *k_4p_* for the 5′-NTR exchange (eq. 7, 8 and 9), corresponding to the rates of the minus- and plus-strand initiation and synthesis, respectively (for reference, see figure 2C). Comparison of our prediction with experimental measurements demonstrated that in both cases the model qualitatively agreed with the experimental data. Consequently, upon refitting of these parameters to the new data, the model was capable of quantitatively describing the perturbed replication kinetics (figure 4C). Furthermore, the model correctly predicted the impact of the respective NTR-exchanges onto the ratio of plus- to minus-strand RNA at the steady state (figure 4D). Predictions for both NTR-exchanges were in close quantitative agreement with our previously published experimental observations, which showed an 8.7∶1 (simulation 9.0∶1) ratio between plus- and minus-strand for the wildtype, 16.1∶1 (11∶1) for the 3′-NTR-chimera, and 4.7∶1 (4.8∶1) for the 5′- chimera [22].

Taken together, our model was able to correctly infer the effects of targeted interventions at different steps of the replication process, including complete replication deficiency, as well as specific inhibition of plus- or minus-strand RNA synthesis, respectively. We therefore conclude that our model provides a realistic description of HCV RNA replication dynamics, and thus can be confidently used to further study such processes *in silico* that are difficult or impossible to address experimentally.

### Replication vesicles are load and choke points of viral replication

**Figure 5:**
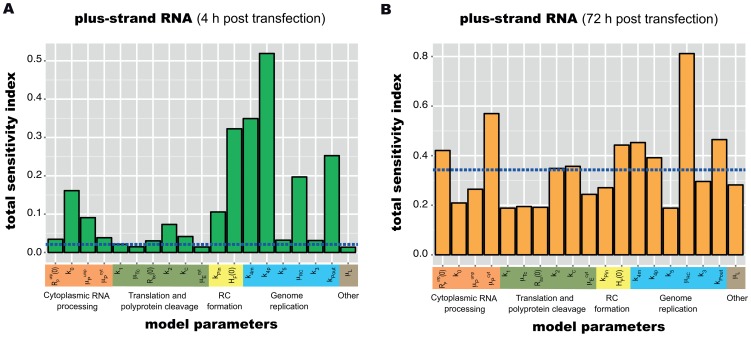
Global sensitivity analysis of the replication model. Sensitivity analysis was performed using the extended Fourier Amplitude Test (eFAST) at (A) 4 hours and (B) 72 hours. Shown are eFAST total order sensitivity indices for plus strand RNA; sensitivities for minus strand RNA and viral protein can be found in supplementary figure S4. The dotted blue line indicates the level of a negative control parameter that does not occur in any of the equations. Sensitivities lower or equal to this negative control should not be considered significantly different from zero [39].

Having such a comprehensive and accurate model at hand, we proceeded by applying it to concrete problems in the field of HCV research. The first question we addressed was which sub-steps of HCV RNA replication would be most susceptible to targeted interference. Such processes are potentially attractive targets for the design of new DAAs against HCV. To find out which step in the replication cycle has the biggest impact on the resulting RNA and protein levels, we assessed the relative sensitivity of replication towards alterations of reaction rates in the model. To account for the two clearly discernable phases of replication – the highly dynamic establishing phase and the steady-state phase – we performed a global sensitivity analysis using the extended Fourier Amplitude Sensitivity Test (eFAST) [39], [40] at an early (4 h) and at a late (72 h) time point. We separately assessed the sensitivities of plus-strand RNA, minus-strand RNA as well as protein levels towards individual and simultaneous changes of 16 rate constants and the three initial values (figure 5 and supplementary figure S4).

For the establishing phase of replication, this analysis showed that the most influential processes are the polyprotein translation (rate *k_2_*), the export rate of RNA into the cytoplasm (rate *k_Pout_*) and the efficiency of plus- (rate *k_4p_*) and minus- (rate *k_4m_*) strand RNA synthesis inside the replication compartment, respectively (figure 5A). As one would expect, alterations in *k_2_* mainly influence the amount of viral protein (eq. 4 and 6) and only to a lesser degree viral RNA (eq. 2 and 3), whereas *k_4m_* mainly affect RNA species (eq. 7, 8 and 9). *k_4p_* and *k_Pout_* in contrast strongly influence RNA and protein concentrations (eq. 8, 9, 10 and 11). Further important steps are the initial “processing” of the transfected RNA (rate *k_0_*), since this determines at what time and to what extent RNA is available for translation, as well as the RNA degradation rate *μ_RC_* inside the replication compartment. The availability of viral RNA for rapid genome replication and the replication process inside the membranous web itself are therefore key determinants of the initial replication dynamics and thus the efficiency of infection, and consequently constitute a very attractive target for anti-viral drugs. Interestingly, the rate of polyprotein translation (eq. 4) naturally has a big impact on viral protein concentration, but only a fairly restricted influence on RNA levels. Furthermore, the cleavage rate of nascent viral polyprotein (eq. 4 and 5, rate *k_c_*) only very mildly impacts replication dynamics.

A profoundly different pattern can be observed for the steady state phase. The single most influential parameter determining viral RNA and protein levels was found to be the degradation rate of viral RNA inside the replication vesicles *μ_RC_* (eq. 7 to 11), while most other parameters showed no significant sensitivities (figure 5B and supplementary figure S4). However, it is virtually impossible to influence this parameter by cellular (e.g. innate immune) or pharmacological intervention (except by physical destruction of the membranous structures), therefore making inhibition of viral replication particularly cumbersome once the steady state has been established. Taken together with the results from the early phase, these analyses suggest a key role of the replicative compartment for a successful establishment and maintenance of infection.

### Replication vesicles attenuate exponential RNA replication and balance protein translation and RNA replication

**Figure 6:**
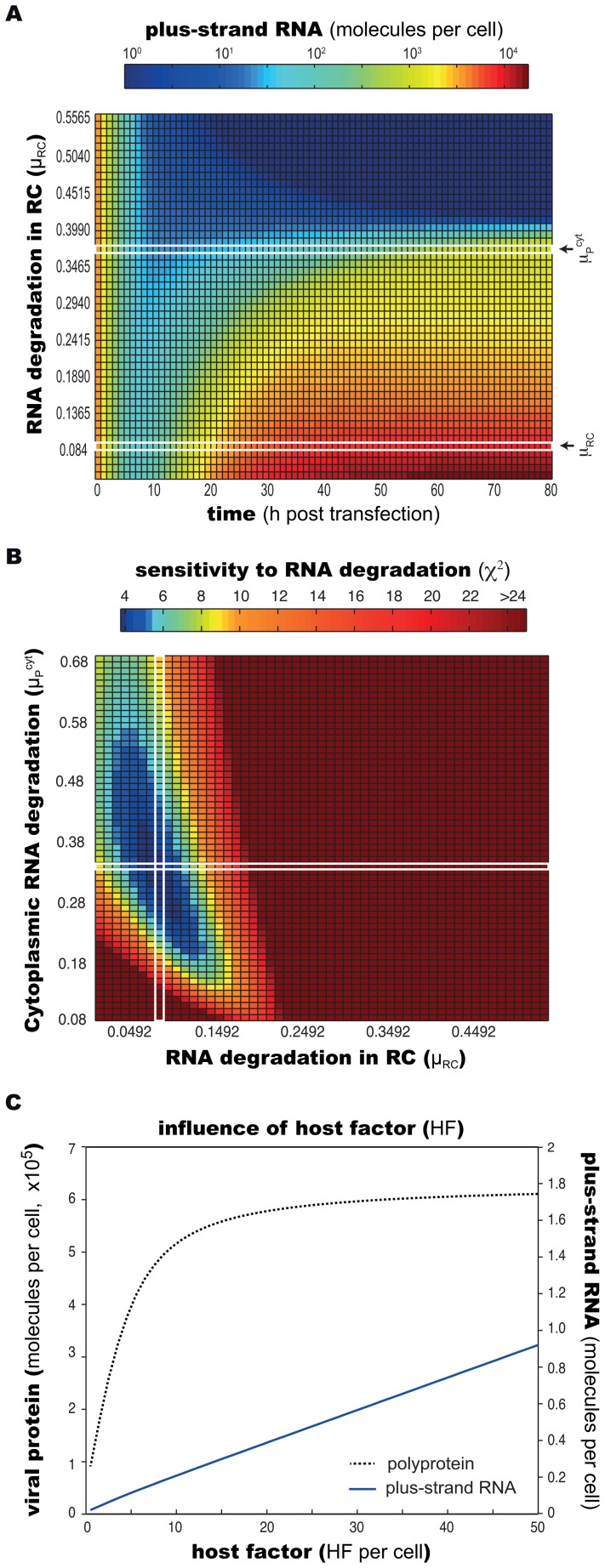
Analysis of the importance of a distinct replicative compartment (RC). (A) Protective effect of replication vesicles: replication dynamics (plus strand RNA shown) at different degradation rates (*μ_RC_*) of viral RNA inside of the replicative compartment (RC). Actual values for *μ_RC_* and *μ_P_^cyt^* obtained from model calibration are marked in the figure. Different degradation rates are depicted on the y-axis, resulting time courses for positive strand RNA molecules are color-coded along the x-axis. At *μ_RC_* = *μ_P_^cyt^*, viral RNA replication becomes unstable, and efficient replication cannot be sustained. (B) The plot shows the resulting sum of residual squared errors (*χ* ^2^) when simultaneously varying the degradation rates *μ_RC_* and *μ_P_^cyt^*. The plot shows that *χ* ^2^ increases over five-fold when *μ_RC_* and *μ_P_^cyt^* attain similar magnitudes. (C) Effect of host factor (*HF*) expression levels on the steady state levels of viral RNA and protein. Plus-strand RNA steady state levels (red line) respond linearly to concentration changes of *HF* in the range of 1–100 *HF* “molecules”. Viral polyprotein levels (blue line) show a bi-phasic steady state behavior with an exponential response for up to approx. 20 *HF* “molecules”, showing saturation thereafter. Note that *HF* is a hypothetical species likely comprising different host cellular proteins and/or processes; “molecules” therefore does not reflect physical molecule numbers.

In the light of the above findings, pointing to a central role of the membranous web within the RNA replication cycle, we further studied the underlying molecular functions of this compartment. For one, we assessed the importance of its protective character onto the dynamics of viral genome replication. Model fitting led to a more than 4-fold lower RNA degradation rate within the replication compartment (*μ_RC_*) as compared to RNA degradation in the cytoplasm (*μ_p_^cyt^*, see table 1). To simulate the effect of less stringent protection of the RNA inside the RC, we then deliberately increased its degradation rate (*μ*_RC_) and calculated the resulting levels of plus strand RNA over time (figure 6A). This analysis showed that the degradation rate inside the replicative compartment inversely correlated with the amount of RNA produced at any given time. Interestingly, this correlation was not continuous, exhibiting a threshold of productive RNA replication, constituting a “cliff”, crossing of which prevented the establishing of a (non-zero) steady-state and effectively killing off viral replication (figure 6A, dark blue area, see also supplementary figure S5). This highly instable region with very low (or zero) RNA copy numbers, strikingly, was reached once degradation inside the RC (*μ_RC_*) was approximately equal to the degradation rate in the cytoplasm (*μ_p_^cyt^*). Our model therefore predicts that the viral RNA must be protected from active degradation during replication in order for HCV to maintain robust persistent replication. While it is virtually impossible to reproduce the above findings in a biological experiment (i.e. increasing RNA degradation inside the replicative compartment), previous *in vitro* data actually showed that viral RNA in the cell, particularly the minus-strand, is highly resistant to nuclease treatment [10], implying that indeed degrading enzymes cannot enter the replication vesicles. Moreover, in inhibitor studies, ongoing HCV replication was blocked by interferon or a pharmacologic NS3/4A inhibitor, leading to rather slow decrease of RNA with a half-life of 12–20 h [41], [42], most likely representing a slow degradation of replication vesicles. In good agreement with these studies, our model predicts a half-life for RNA inside the replicative compartment of 12 h (rate *μ_RC_* = 0.08 h^−1^), whereas RNA transfected into the cytoplasm decayed with a half-life of approximately two hours in the experiments using a replication-defective replicon (see figure 4A). Experimentally very hard to address, however, is the degradation rate *μ_p_^cyt^* of cytoplasmic HCV RNA generated through replication that might exhibit a different folding or be bound by other proteins as compared to transfected RNA. Yet, it appears highly likely that this degradation rate would more closely match the rate of degradation of transfected, cytoplasmic RNA rather than that of RNA within the membranous replicative environment. In keeping with this plausible assumption, our model predicts a half-life for newly synthesized cytoplasmic RNA of approximately 165 min (*μ_p_^cyt^* = 0.363 h^−1^). Although model estimations for both, *μ_p_^cyt^* and *μ_RC_*, exhibit a rather broad confidence interval, simultaneous modification of both parameters shows that *μ_RC_* needs to be substantially lower than *μ_p_^cyt^* in order to explain the observed kinetics (figure 6B, dark blue area). In terms of viral protein, Quinkert and colleagues showed that in contrast to RNA, only a small fraction (<5%) of NS5B molecules is protease resistant [10]. In line with these observations, our model predicts that the vast majority of viral protein remains in the cytoplasm.

**Table 1:** Parameter estimates obtained from model calibration.

Rate constant	Definition	Rate constant	90% confidence interval	Reference
*k_0_*	Processing rate of transfected positive-strand RNA	0.00415 h^−1^	(1.07e-3, 1.61e-2)	
*k_1_*	Formation rate of translation complex	1 h^−1^ molecule^−1^	Fixed after sensitivity/identifiability analysis	
*k_2_*	Polyprotein translation rate	100 h^−1^	Experimentally observed	[24]
*k_c_*	Polyprotein cleavage rate	1 h^−1^	Fixed after sensitivity/identifiability analysis	
*k_Pin_*	Formation rate of the plus-strand replicative intermediate complex	9.04e-6 h^−1^ molecule^−2^	(3.85e-7, 2.12e-4)	
*k_Pout_*	Transport rate of nascent plus-strand RNA into cytoplasm	0.307 h^−1^	(0.167, 0.538)	
*k_3_*	Formation rate of the plus-strand replicative intermediate complex from within the RC	10^−4^ h^−1^ molecule^−1^	Fixed after sensitivity/identifiability analysis	
*k_4m_*	Minus-strand RNA synthesis rate	1.7 h^−1^	Experimentally observed	[23], [77], [78]
*k_4p_*	Plus-strand RNA synthesis rate			
*k_5_*	Formation rate of the minus-strand replicative intermediate complex	10 h^−1^ molecule^−1^	Fixed after sensitivity/identifiability analysis	
*μ_P_^unp^*	Degradation rate of unprocessed plus-strand RNA	0.754 h^−1^	(0.510, 1.11)	
*μ_P_^cyt^*	Degradation rate of processed plus-strand RNA	0.363 h^−1^	(0.168, 0.783)	
*μ_Tc_*	Degradation rate of translation complex	0.181 h^−1^	(0.0841, 0.392)	
*μ_E_^cyt^*	Degradation rate of NS5B protein	0.06 h^−1^	Experimentally observed	[76], [81], [82]
*μ_RC_*	Degradation rate of RNA and E in the replication compartment	0.0842 h^−1^	(0.0193, 0.366)	
*μ_L_*	Degradation rate of luciferase	0.35 h^−1^	Experimentally observed	[79], [80]
*HF_high_(0)*	Initial values for activated host factor in high permissive cells	48 molecules	(11, 215)	
*HF_low_(0)*	Initial values for activated host factor in low permissive cells	4 molecules	(1, 14)	
*R_ibo_(0)*	Total ribosome complexes	628 molecules	(68, 5810)	
*_fScale_*	Scaling factor for Luciferase polyprotein marker	2160	(474, 9870)	

Parameter estimates and confidence bands were obtained using multiple shooting, simultaneously fitting the model to the data from Huh7-Lunet and Huh-7 lp cell lines.

Another important question, which can hardly be addressed experimentally, is the possibility of re-initiation of minus-strand synthesis inside the replication vesicle. While theoretically it is feasible that the replicative machinery re-initiates minus-strand synthesis on newly produced plus-strands inside the replication compartment (eq. 7, second to last term), the alternative hypothesis is that such an initiation event can only happen in *cis* upon translation in the cytoplasm (see also section on model development above). In fact, when analyzing the calibrated model, we found that the rate constant for this reaction (*k_3_* in eq. 7, see figure 2C for reference) needed to be close to zero (<10^−4^ h^−1^*molecules^−1^) to fit the experimental data, and the concentration of “active” polymerase (*E*) was severely limiting the rate of RNA synthesis during the initial dynamic phase. This resulted in an extremely low efficiency of internal re-initiation, implying that most or all of the newly synthesized viral plus-strand RNA is exported to the cytoplasm, from where it must be re-imported for further rounds of RNA replication to occur. Hence, our model supports the notion that negative-strand initiation is very different from plus-strand initiation in that it most likely depends on actively translated RNA with the required NS proteins, mainly NS5B, being supplied in *cis*.

The observed relative shortage of active polymerase within the replication vesicles and the lack of internal re-initiation consequently prevents an exponential amplification of the viral RNA within the replicative compartment. Replication vesicles thus attenuate the rate of viral replication by limiting the availability of the factors required for minus-strand initiation. At the same time, depending on the export rate of newly synthesized plus-strand RNA from the replication vesicles (*k_pout_*), they can also exert tight control over protein translation. Newly synthesized RNA can either be exported to the cytoplasm where it can be used for another round of protein translation (or, in an actual infection setting, the assembly of new viral particles), or it accumulates within the replication vesicles; there, however, it cannot be used as a template for minus strand synthesis due to the above described reasons.

Taken together, the development of a membranous replication compartment, by physically separating production of new protein (translation) and the generation of new RNA (replication), therefore constitutes an important additional level of control over the virus' replication kinetics. This high degree of controllability might be one reason for the evolutionary success of membranous replicative structures, as basically formed by all positive strand RNA viruses. In case of HCV, it allows for sustained low-level replication as is required for the establishment of persistence, mainly by restricting availability of the required proteins within the replicative compartment.

### Different processes are limiting RNA replication in high and low permissive cells

Particularly for a persistent virus, tight control over its own replication is essential in order to not overwhelm its host cell and thereby kill it [13]. As we have learned above, the distinct replication compartment plays a central role in this self-limitation. Consequently, we therefore studied, which processes in turn regulate the formation of replication vesicles and eventually lead to the establishment of a steady state. The host factor (*HF*) in our model has been found to be a requisite for the attainment of a steady state and its concentration was a determinant regulating plateau levels of viral RNA and protein between the two differently permissive cell lines. For that reason, we now systematically assessed the impact of different availabilities of *HF* onto steady-state levels of viral RNA and protein. For HCV RNA levels, this analysis showed a linear correlation with *HF* concentrations in the range tested: the more abundant *HF* was, the more RNA replication took place. Interestingly, however, polyprotein levels exhibited a saturation behavior, reaching a plateau for *HF* concentrations above 20 “molecules” (note that *HF* is a virtual species, so “molecules” is an arbitrary unit) (figure 6C). To understand this nonlinear dependence of viral protein on *HF* levels, we analyzed the model under conditions of varying *HF* amounts and found that this saturation stems from different factors being limiting for increasing *HF* levels: in low permissive cells (featuring low *HF* concentrations of around 4 “molecules”), *HF* availability is limiting the formation of replication vesicles (eq. 7). Therefore, overall RNA concentrations remain relatively low, leaving polyprotein production at a low but steady level; here, RNA in the cytoplasm is the rate limiting factor for protein translation. In high permissive cells (featuring high *HF* levels of around 50 “molecules”), in contrast, rapid formation of replication vesicles occurs with an associated rapid increase in viral RNA levels. However, ribosome availability (*R_ibo_*) then becomes limiting for protein translation (eq. 3), explaining the plateau seen for viral protein concentrations (figure 6C). Accordingly, the ratio between viral protein (i.e. luciferase) and plus-strand RNA is lower in the steady state in high permissive cells. This is well in line with the experimental data (figure 1, compare B and C).

Interestingly, these findings suggested that the actual mechanisms governing the establishing of the steady state in low permissive and high permissive cells are different. While in low permissive cells the formation of replication vesicles is the limiting step due to a lack of host factor *HF*, surprisingly the host translation machinery is the bottleneck in high permissive cells.

### Transcriptional profiling of different host cells identifies genes correlating with cellular permissiveness for HCV RNA replication

**Figure 7:**
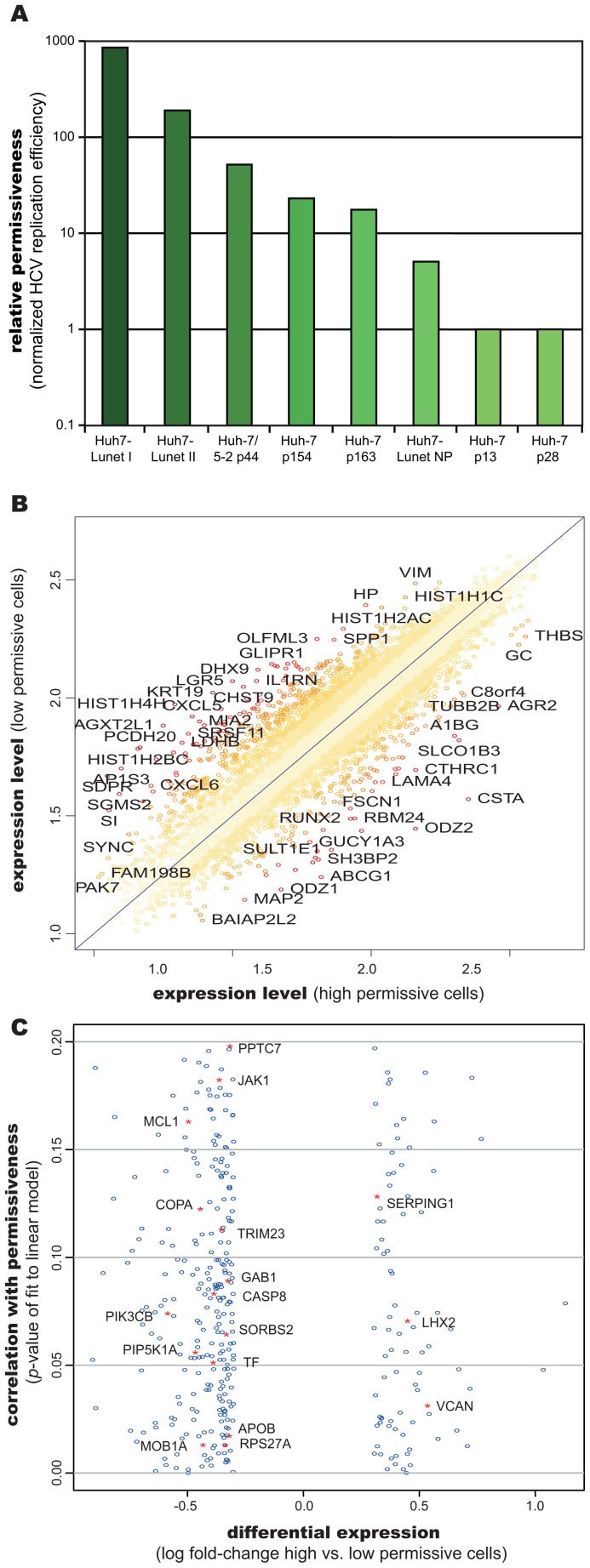
Gene expression profiling of differently permissive Huh-7 cells. (A) Relative permissiveness for HCV replication of eight different Huh7 derived cell lines. Permissiveness was normalized to Huh-7 p28 cells. (B) Scatterplot of host gene expression in high permissive Huh7-Lunet versus low permissive Huh-7 lp cells. Off-diagonal elements are differentially expressed and are potential candidates underlying the difference in replication efficiency. Colors encode the distance from the diagonal. A selection of strongly differentially expressed genes is labeled with gene symbols. (C) Eight different cell lines with different replication permissiveness (see panel A) were used, and replication efficiency was correlated with host gene expression. A linear model was fitted to predict replication permissiveness from gene expression data, and goodness of fit assessed using ANOVA. Shown are resulting *p*-values, plotted over the log- fold-change of expression between Huh7-Lunet and Huh-7 lp cells. Shown are genes with *p*-values<0.2 and a log-fold-change of more than 0.3 or less than −0.3. Seventeen genes that were previously shown to be functionally linked to HCV replication or to directly interact with viral proteins are highlighted in red and labeled.

As differential abundance of the host factor (or host process) *HF* in our model sufficed to explain the observed difference in HCV replication dynamics between high and low permissive cells, it was intriguing to identify the biological nature of this factor. For that purpose, we set out to compare gene expression profiles of Huh-7 cells of different passage number or clonal origin that we had found to exhibit substantially different permissiveness for HCV RNA replication [22], [30] (figure 7A). We performed full-genomic cDNA microarray (Affymetrix HGU133plus 2.0) analysis in eight such Huh-7 derived cell lines, including the above used Huh7-Lunet and low passage (lp) Huh-7 cells. Figure 7B shows a scatterplot of the normalized gene expression values for these two cell lines. Assuming a direct correlation between permissiveness and the expression of the host factor *HF* as suggested by the above analysis (compare figure 6B), we fitted a linear model of each gene's expression level to the observed replication efficiencies in all eight cell lines. By this, we could assess how well each individual gene predicts replication efficiency over the full set of cells. On these data, we then carried out an analysis of variance (ANOVA) to identify genes whose expression profiles correlated significantly with replication efficiency. Figure 7C shows the resulting p-values over the degree of differential expression (as log fold-change) between Huh7-Lunet and Huh-7 lp (see also supplementary table S1). We could identify 355 genes, whose expression levels correlated with permissiveness (p<0.2) and which exhibited a difference in expression levels of more than 23% (log fold-change >0.3 or <−0.3) (figure 7C and supplementary table S2). We then subjected these potential *HF* candidates to bioinformatics analyses in order to identify host cellular processes or pathways, which are over-represented among those genes (supplementary tables S3 and S4). These analyses mainly identified metabolic processes such as lipid metabolism and cell growth and proliferation, which is in line with the notion of HCV RNA replication requiring proliferating cells for efficient replication, at least in Huh-7 cells [43], and numerous reports on its requirement on lipid biosynthesis (reviewed in [44]).

While the number of potential *HF* candidate genes was too large to be functionally validated individually within this study, we surveyed previously published data on HCV host factors, including a manually curated database of HCV-host interactions (VirHostNet [45]) as well as large-scale siRNA-based screens [46], [47], [48], [49]. Whereas such high-throughput approaches exhibit very high false-negative rates [50] and therefore are not suited to exclude *HF* candidates from our analysis, their false-positive rate is very well controlled and the identified hit genes are highly reliable. Using these data, we could in fact identify 17 of our *HF* candidates to be implicated with HCV (table 1; marked in red in figure 7C). Six of these genes (JAK1, LHX2, PIP5K1A, RPS27A, PPTC7 and COPA) were found in siRNA-mediated approaches to directly influence HCV replication, as would be expected for a limiting host factor. Five genes (TF, VCAN, TRIM23, SORBS2 and MOBK1B) were identified in a large-scale yeast-two-hybrid based interaction screening [51] to interact with at least one HCV protein (interaction partner listed in table 1). This, however, does not necessarily indicate that the interaction is essential for RNA replication. On similar lines, six further genes (MCL1, SERPING1, CASP8, PIK3CB, GAB1 and APOB) were previously reported to interact with specific HCV proteins in individual studies. Interestingly, most of them (MCL1, CASP8, PIK3CB and GAB1) were implicated with a modulation of apoptosis and cell survival/proliferation, supporting our above analysis, in which “cell growth and proliferation” was found to be an enriched function among the differentially expressed genes (supplementary tables S3 and S4).

Based on our model prediction of a limiting host factor/process involved in the formation of functional replication compartments and utilizing our transcriptomic analysis of differently permissive cells, further studies should be devised aiming to delineate the exact nature of this factor or process. Identification of a cellular function that is essential for HCV replication but naturally limiting in certain cell lines would be very intriguing in terms of pinpointing novel targets for anti-HCV therapy. Such a factor would promise to be inhibitable without critically affecting host cell viability, while severely compromising HCV replication efficiency.

## Discussion

### Extended mathematical model precisely predicts HCV RNA replication dynamics in different cells

In the present study, we have developed a mathematical model of the intracellular steps of HCV replication. In contrast to previous models [24], [25], [26] we were not only interested in studying steady state replication in stable replicon cell lines, but specifically addressed the highly dynamic initial phase after RNA genome delivery into the host cell. We therefore performed quantitative, time-resolved measurements of viral protein translation as well as strand-specific viral RNA concentrations in two distinct Huh-7 derived cell lines, exhibiting a vastly different permissiveness for HCV RNA replication [32]. With this data, we tried to re-calibrate the most comprehensive HCV replication model available to date [24], but found that the model was not capable of explaining the observed dynamics and ensuing steady state simultaneously. We therefore modified and extended that model by accounting for the “naked”, unprotected nature of the initially transfected *in vitro* transcribed RNA and by updating of the formation step of the RC and the initiation of negative strand RNA synthesis to the current biological understanding of this process. Under steady state conditions, as studied by previous models, equilibrium of the viral replication machinery with static ratios between cytosolic viral RNA and NS proteins has been achieved. Therefore, in the model by Dahari and colleagues [24], uptake of viral RNA and protein into the replicative compartment could be described by simple first order import reactions. In our setting, however, concentrations for replication competent viral RNA and NS proteins start from zero and grow dynamically in the course of the experiment. Hence, simple first order import reactions do not suffice if the uptake depends on the abundance of more than one species, which is highly likely given biological evidence. Accounting for the above described *cis*-requirement for initiation of productive replication complexes [35], [36], [37], which means that an RNA molecule can be used as a template for replication only by an NS5B molecule having been translated from that very RNA, we solely allowed a complex of actively translated plus-strand RNA (i.e. translation complexes *T_C_*) and cytosolic NS proteins (*E^cyt^*) to be taken up into the RC.

While these model extensions greatly enhanced the fitting quality to the data of a single cell line, we then identified that step in the model, at which an altered kinetic rate could explain the dynamics found in the second cell line as well. For this purpose, we tested a series of hypotheses, fitting the model simultaneously to the two differently permissive cell lines and allowing only those parameters to differ that would be influenced by the host cell in the respective hypothesis. By this approach, we could exclude various processes, e.g. differences in translation efficiency, altered cytoplasmic RNA degradation or different RNA synthesis rates within replication complexes. It is also biologically plausible, that these processes do not differ between the two examined Huh-7 cells lines and therefore cannot explain the observed differences in permissiveness; both, translation and RNA degradation have been shown before to be comparable across different Huh-7 cells [30], and the polymerization rate of the HCV RdRP NS5B is unlikely to depend on host factors (other than ribonucleotides). In principle, a combination of several such processes might be able to explain the observed behavior; however, following Occam's razor, we considered the simplest solution to be the most likely one. Eventually, we identified the formation process of replicative vesicles to be the best suited step in the model, altering the rate of which sufficed to fit the model to measured data from either cell line. We then introduced a host factor (*HF*) as a new species in our model, and required viral RNA (in the form *T_C_*) and NS protein (*E^cyt^*) to form a complex with it in order to allow for the initiation of negative strand RNA synthesis and the formation of the RC. Assumption of different concentrations of this host factor then was sufficient to very accurately explain the differences in RNA replication permissiveness in the two cell lines. This final model therefore completely satisfied all experimental observations and could also correctly predict the effects of targeted perturbations during extensive validation experiments.

### Mathematical HCV replication model defines optimal targets for pharmacologic intervention

We then used the calibrated and validated model to further study individual steps of the viral lifecycle. Sensitivity analysis was applied to pinpoint the most influential steps, perturbation of which would lead to the greatest impact on replication dynamics and yield. A very interesting first finding was that once steady state replication has been reached, the system proved to be relatively robust towards perturbation of individual sub-steps of replication. The degradation rate of RNA inside the RC was the most sensitive parameter under these conditions, and had a significantly higher influence than all other rates. This parameter, however, can hardly be influenced biologically or therapeutically. Very likely, this robustness is key to HCV's prevailing in the face of cellular stress- and innate immune responses [52], [53], [54], [55]. The actual mechanistic basis of this remarkable robustness so far remains elusive.

In contrast, at an early time point after introduction of HCV genomes into the cell, the system was found to be substantially more fragile with respect to the number of sensitive parameters. This suggests that therapeutic intervention with HCV replication by DAAs would be most efficient in newly infected cells, emphasizing the potential of such drugs for the prevention of reinfection upon liver transplantation. The processes found to be most sensitive during the early phase of replication were polyprotein translation as well as the RNA polymerization rate of NS5B. Of note, polyprotein cleavage by the viral NS3/4A protease was surprisingly little influential. This, however, has been described before, e.g. in a study examining the role of cyclophilin A for HCV replication [56]. In that study, viral mutations conferring resistance to the cyclophilin A inhibitor Alisporivir (Debio-025) were shown to significantly affect the efficiency of polyprotein cleavage without notably affecting RNA replication of the replicon [56]. This could raise some concern about the first (very recently) approved direct acting antivirals for HCV, the NS3/4A inhibitors Telaprevir and Boceprevir [57]: on the one hand, they need to exhibit an extremely high potency of inhibition in order to suppress HCV RNA replication efficiently. On the other hand, there should be comparatively little restrictions to the development of escape mutations rendering NS3/4A resistant to the compounds, owing to the relatively small effect on replication dynamics even in a case where the mutation functionally lowers protease activity as it is predicted by our model. Simply put, the virus can effectively buy itself out of pharmacologic inhibition at only modest fitness costs, and in fact, at least for the first generation of protease inhibitors, this is indeed the case [58], [59]. In contrast, according to our model analysis, HCV should be far more sensitive towards inhibition of the NS5B polymerase activity. In line with this prediction, an NS5B inhibitor (HCV-796) yielded a significantly faster and stronger response when directly compared to a very potent protease inhibitor (BILN 2061), both dosed at the same multiples of their respective EC50s [60]. This difference in efficaciousness could even get potentiated when considering the development of escape mutations. Particularly for nucleoside/nucleotide analogues, which target the catalytically active center of NS5B, all so far observed resistance mutations have a negative influence on its polymerase activity [61]. Based on our model, however, lowering NS5B activity is predicted to have a pronounced impact on overall replication efficiency, thereby substantially increasing the fitness costs for such escape mutations. In fact, despite being “genetically easy” (i.e. single nucleotide exchanges suffice) such resistance mutations against nucleotidic inhibitors have been shown to be of negligible clinical relevance due to their extraordinarily strong impact on replication efficiency [62]. In general, we want to note that a modeling approach as ours can help in estimating and understanding the sensitivity of HCV replication upon (e.g. pharmacologic) inhibition of a particular step in the life-cycle. It cannot, however, generally predict the development of resistance mutations, as the actual number and position of nucleotide/amino acid exchanges required for resistance eventually determine the likelihood of their occurrence and their fitness-cost, respectively.

### Steps of RNA replication and involvement of host factors

One simplification that we accepted in developing the model is that the formation of the membranous vesicles is modeled as one step (eq. 7) together with the formation of the actual replicase complexes (i.e. the initiation of negative strand RNA synthesis). This is owing to a lack of an experimental handle for the discrimination of “productive” from empty or non-functional vesicles. In fact, it has been shown that the vesicular membrane structures are formed by viral NS protein also in the absence of RNA replication [6], [63]. Therefore it seems likely that initiation of RNA synthesis will depend on the formation of membrane alterations, but still represents a distinct step in the formation of an active replication site. However, in this two-step scenario, membranous vesicles would form based on the concentration of cytosolic NS proteins (*E_cyt_*) and a host factor (*HF*), and replication complexes (*R_ip_*) would mainly depend on *T_c_* (and possibly *E_cyt_* and *HF*) and the availability of vesicles. In effect, formation of productive replicative vesicles would again depend on those three species, *T_C_*, *E_cyt_* and *HF* and should in principle be compatible with our simplified one-step model.

On similar lines, for reasons of simplicity, our model considers only one single, large replication compartment. This assumption is clearly not correct, as numerous sites of virus induced convoluted membrane structures have been observed in HCV replicating cells [7] and each cell holds approximately 100 negative strand RNAs (i.e. markers for productive replication complexes) on average [10]. However, the approximation with a single large replicative compartment should be adequate provided the real number of vesicles is large enough for formation or loss of individual vesicles not to lead to significant sudden changes of viral RNA and protein availability in the cytoplasm. As measurements of replication are technically limited to bulk assessments and cannot probe individual vesicles, for the time being this point cannot be addressed more adequately. Similarly, there might also be (and likely is) heterogeneity among cells in terms of kinetics and absolute numbers. Also here, probing individual cells for plus and minus strand RNA as well as for polyprotein production is almost impossible with today's technology, and consequently, our model represents an approximation of the average cellular behavior in a larger population of cells.

Curiously, a central result of our study was the conclusion that the assumption of a key host factor was essential to fit our model to the dynamics of RNA replication. This factor was important to explain RNA replication in Huh-7 cells, but might not be as limiting in other HCV permissive cells, e.g. primary human hepatocytes. Moreover, in a physiological setting, restrictions in other steps of the viral life cycle, e.g. sub-threshold receptor levels during entry [64], [65] or a limitation in the apolipoprotein system required for particle secretion [66] might play critical roles as well. Importantly, also the innate immune response (and on a larger time-scale also the adaptive one) poses severe restrictions on viral replication via effector genes, whose molecular identity and functions have only recently begun to be identified [67], [68]. These influences would need to be included in a future, fully comprehensive model of HCV replication. For our present model, based on Huh-7 cells, however, we have so far neglected any impact by the innate immune system, as we could previously demonstrate that presence or absence of functional immune recognition of HCV by the (Huh-7 derived) host cell does not have a measurable effect on its permissiveness [32].

Still, for RNA replication in this single most important cell culture system for HCV, we found a limiting host function involved in the formation of the replication compartment to be crucial to explain the observed replication kinetics. The molecular function of this host factor is still unclear; one or more cellular proteins could be involved, taking part in the formation of the membrane alterations or in the initiation of RNA synthesis. Even a more general condition such as stress tolerance could serve as the host requirement proposed by our model. Since this host factor(s)/condition(s) *HF* was sufficient to model the varying RNA replication efficiencies in different Huh-7 populations, we performed gene expression profiling to identify genes potentially defining permissiveness. While our analysis identified 355 genes, whose expression correlated with the degree of permissiveness of the respective cell line, there were no single factors or well-defined pathways that stood out significantly. In order to test the limiting nature of these identified factors for HCV RNA replication, one would have to individually overexpress those genes in low permissive cells and assay for an enhancement in HCV replication. Whereas this was beyond the capacity of our current study, we made use of extensive publicly available data on cellular interaction partners of HCV (VirHostNet [45]) and high-throughput RNAi-based knock-down studies [46], [47], [48], [49] in order to recognize genes that had been implicated with HCV before. This approach identified 17 cellular genes whose expression levels on the one hand correlated well with permissiveness for HCV replication, and that, on the other hand, were either reported to at least interact with an HCV protein, or were shown to have a direct impact on HCV replication upon knock-down (table 2). While for this small sub-set of genes a reliable functional link to HCV could therefore be established, we cannot exclude any of the remaining differentially expressed genes as potentially crucial host factors for HCV; this is true even in spite of a virtually genome-wide coverage of the published screening studies, as such approaches are characterized by extremely high false-negative rates [50]. Therefore, comprehensive future studies need to exploit the information contained in our transcriptomic analysis, systematically testing those host factors for an impact on HCV replication that most significantly correlated with permissiveness.

**Table 2:** Established HCV host factors identified in transcriptomic analysis.

Gene Symbol	Gene ID	Gene Name	Previous Hit (Reference)	Interaction partner	log2 fold change HP/LP	p-value
MCL1	4170	Myeloid cell factor 1	[45], [90]	Core	−0.4980	0.1632
TF	7018	transferrin	[45], [51]	E2	−0.3918	0.0514
VCAN	1462	versican	[45], [51]	NS3	0.5329	0.0315
TRIM23	373	tripartite motif-containing 23	[45], [51]	NS3	−0.3551	0.1132
SERPING1	710	serpin peptidase inhibitor, clade G (C1 inhibitor), member 1	[45], [91]	NS3	0.3157	0.1285
CASP8	841	Caspase 8	[45], [92]	NS3	−0.3883	0.0835
PIK3CB	5291	phosphoinositide-3-kinase, catalytic, beta polypeptide	[45], [93]	NS5A	−0.5875	0.0743
SORBS2	8470	sorbin and SH3 domain containing 2	[45], [51]	NS5A	−0.3349	0.0646
GAB1	2549	GRB2-associated binding protein 1	[45], [94]	NS5A	−0.3282	0.0895
APOB	338	Apolipoprotein B	[45], [95]	NS5A	−0.3219	0.0175
MOBK1B	55233	MOB1, Mps One Binder kinase activator-like 1B (yeast)	[45], [51]	NS5A, NS5B	−0.4355	0.0133
COPA	1314	coatomer protein complex, subunit alpha	[47]		−0.4471	0.1227
PPTC7	160760	PTC7 protein phosphatase homolog (S. cerevisiae)	[48]		−0.3196	0.1982
RPS27A	6233	ribosomal protein S27a	[46]		−0.3388	0.0131
PIP5K1A	8394	phosphatidylinositol-4-phosphate 5-kinase, type I, alpha	[46]		−0.4684	0.0562
LHX2	9355	LIM homeobox 2	[47]		0.4467	0.0708
JAK1	3716	Janus kinase 1	[49], [51]	Core, NS5A	−0.3653	0.1826

Analysis of genes differentially expressed between high and low permissive Huh-7 based cell lines (log-fold change >0.3 or <−0.3 between high and low permissive cells) and correlated with replication permissiveness of 8 cell lines (p-value<0.2). Resulting genes were intersected with published RNAi screening [46], [47], [48], [49] and virus-host protein interaction [45] data as described, yielding a list of 17 host factors that are differentially expressed between the high and low permissive cells, that correlate with replication permissiveness in the eight cell lines used, and that have previously been shown to be associated with HCV infection or replication.

### The role of membrane alterations in regulating RNA replication

Already during model development, but also throughout our model analyses, the formation and function of the membranous replication compartment was found to be crucial for successful viral HCV replication. Previous literature as well as our model analysis imply that membrane alterations serve at least three distinct purposes. For one, they provide a protected environment for RNA replication, shielding this very sensitive process from the host cell degradative machinery as also shown experimentally before [10]. Without this protection, the viral RNA would quickly be degraded, and replication, according to our model, would become highly vulnerable to stochastic effects due to very low molecule numbers. In fact, should cytoplasmic RNA degradation be only slightly stronger than our mean estimate for *μ_p_^cyt^* (but well within its confidence interval), e.g. upon stress or under conditions of an activated immune response, the system would cross a threshold and replication would die off inevitably. Therefore, to compensate for such a lack of protection of the replication machinery, HCV would have to develop a completely different amplification strategy, most likely involving a much higher rate of RNA synthesis in order to maintain sustained replication. This, very likely, would not be compatible with low-level, low profile replication as required for persistence [13]. Secondly, sequestration of viral replicative intermediates, such as double-stranded RNA, into membranous compartments also shields them from recognition by ubiquitous pattern recognition receptors of the intrinsic innate immunity (which, as described above, is neglected by our current model). A third important aspect, however, is the fact that this strict compartmentalization allows for a tight control of viral RNA replication versus protein translation. By limiting the amount of viral and/or host protein inside, the replicative compartment not only protects, but paradoxically also attenuates RNA replication. Presumably, this serves to limit replication to levels sustainable by the cell and permitting low-level persistent replication over a long period of time with very limited detection by the immune system. At the same time, by controlling the amount of newly synthesized RNA released into the cytoplasm, the vesicles indirectly control the amount of protein translation and, in an *in vivo* situation, particle formation, as was also suggested by another modeling approach [26].

We provide the first comprehensive modeling of the entire RNA replication cycle of a positive strand RNA virus, from the onset of RNA replication to steady state levels. However, membranous replication sites are a hallmark of all positive strand RNA viruses with very different replication strategies. In case of HCV the membranous replication compartment seems to have a rather limiting role in virus RNA replication, probably contributing to viral persistence and chronic disease. In contrast, most positive RNA viruses replicate fast, cause acute diseases and are cleared by the immune system (e.g. the closely related flaviviruses such as Dengue or West Nile virus). Interestingly, in the related group of pestiviruses, pairs of viral isolates have been found, replicating either in a non-cytopathic/persistent or in a cytopathic/acute manner [69]. Upon integration of cellular mRNA sequences into their genomes, dramatically enhancing the efficiency of viral RNA replication, these biotypes switch from well-controlled, persistent infection to an aggressively replicating, cytophatogenic phenotype [70]. Also in case of Sindbis virus, cytopathic replication can be switched to persistence by a single point mutation [71]. Both examples demonstrate a tremendous flexibility to adapt the concept of membranous replication compartments to various replication strategies. It would therefore be highly interesting to use our model as a blueprint for modeling replication kinetics of closely related positive strand RNA viruses following a lytic/acute replication strategy, e.g. Dengue virus or West-Nile-virus. Comparing the principles governing replication of such a virus to the here described strategy of HCV could offer a completely new approach to examining– and eventually comprehending– the general requirements allowing viruses to establish chronicity.

### Extending mathematical modeling towards the whole viral replication cycle and systemic spread

Another obvious yet intriguing direction into which our presented modeling approach could be developed, is extending it to comprise the full infectious virus life cycle, including particle production and secretion, receptor binding and cell entry. In fact, two very recent publications studied RNA replication kinetics upon HCV infection [6], [29] and found a dynamic behavior extremely reminiscent of what we describe here for subgenomic replicons: the initially present RNA is rapidly degraded early upon infection and then starts to replicate exponentially at around 6 to 8 hours post infection, which is reflected in both, plus- and minus-strand RNA signals. This similarity to the kinetics observed in our experiments is remarkable, as initial RNA concentrations are about two to three orders of magnitude less in the infection (roughly 1–50 genomes per cell) as compared to our transfections (∼4.000 genomes per cell). The single major difference to the here described situation in a replicon setting is the increasing excess of plus-strand RNA over the minus-strand for late time points (e.g. 50-fold excess at 72 h) which seems to be due to decreasing minus-strand levels, while plus-strand RNA basically maintains a steady-state [29]. It is intriguing to speculate that this phenomenon might reflect partitioning of the plus-strand RNA into translation/replication on the one hand, and particle assembly/genome encapsidation on the other hand. As encapsidated genomes would no longer be available for initiation of new replication complexes, minus-strand RNA levels should consequently decrease over time. In order to adapt our model to an actual infection setting, however, we will need to switch to a stochastic model to deal with extremely low copy numbers of RNA per cell. Such situations can be addressed mathematically using the Gillespie algorithm, provided appropriate single cell measurements are available. The model could then also be extended to describe the extracellular steps of the viral life cycle, up to receptor binding and cell entry, which could finally allow for very precise simulation of viral spread through a population of naïve cells. Such a comprehensive model would be highly valuable to examine and predict the effects of therapeutic intervention with viral entry or release as compared to inhibition of intracellular steps of replication. Even more importantly, it could be suited to finally link our fine-grained molecular model of HCV replication to the very interesting patient-level models of HCV infection and therapy dynamics [14], [72], and thereby open up new avenues to rationally designing novel therapeutic strategies, but also to understanding the effects of molecule-scale events onto the progression of a complex disease.

## Materials and Methods

### Cells and cell culture

All cells were maintained in supplemented Dulbecco's modified Eagle medium (DMEM) as described previously [10]. Huh-7 *low passage* refers to naïve Huh-7 cells, passaged less than 30 times in our laboratory, see also Binder et al. [32]. Huh7-Lunet and Huh-7/5-2 are highly permissive clonal cell lines [32]. Huh7-Lunet NP (unpublished) refers to a derivative of Huh7-Lunet, which is significantly less permissive than its parental cell line.

### HCV constructs and *in vitro* transcription

For kinetic analyses of HCV RNA replication, the genotype 2a (JFH1 isolate) constructs pFKi389LucNS3-3′_dg_JFH (wild-type) and pFKi389LucNS3-3′_dg_JFH/ΔGDD (replication deficient) [73] were used, as well as the NTR-chimeric constructs pFK-I_341_PI-Luc/NS3-3′/JFH1/5′Con (5′-NTR exchange) and pFK-I_341_PI-Luc/NS3-3′/JFH1/XCon (3′-NTR exchange) [22]. Permissiveness of cell lines was assessed using a genotype 1b (con1) replicon, using the plasmid pFK-I_341_PI-Luc/NS3-3′/Con1/ET/∂g. *In vitro* transcription of HCV replicons was performed as described previously [22], [30]. Briefly, plasmid DNA was purified by phenol/chloroform extraction and transcribed with 0.9 U/µl T7 RNA polymerase (Promega). RNA was then purified by DNase (Promega) digestion, extraction with acidic phenol and chloroform and room temperature isopropanol precipitation. RNA concentration was determined spectrophotometrically and integrity was confirmed by agarose gel electrophoresis.

### Electroporation of HCV RNA and luciferase assay

Cells were transfected with *in vitro* transcribed HCV RNA by electroporation as described previously [22]. For determination of host cell permissiveness (figure 7), 5 µg of RNA were used for electroporation and cells were seeded into 6-well plates (1/12 electroporation per well). Samples were lysed at 4, 24, 48 and 72 h post transfection and stored at −80°C until measurement of luciferase activity. For time resolved quantitation of HCV replication, 4×10^6^ cells were transfected with 10 µg of HCV RNA, corresponding samples were pooled and cells were seeded into 6-well plates for luciferase assays as described above or into 10 cm cell-culture dishes at a density of 4×10^6^ cells per plate (2×10^6^ cells/plate for time points 48 h and 72 h) for RNA preparation and Northern blotting. For the 0 h RNA sample, 4×10^6^ cells were washed twice with DMEM directly after electroporation, pelleted and lysed in guanidinium isothiocyanate. Other samples were lysed at the indicated time points (2, 4, 8, 12, 18, 24, 48 and 72 h) and lysates were stored at −80°C until further processing.

For determination of HCV replication by luciferase activity measurement, all samples of one experiment were frozen at −80°C upon harvesting and thawed simultaneously prior to luciferase detection. Measurements were performed as described in Binder et al. [22], with all samples measured in duplicate. Luciferase activity was normalized to the input activity assessed at 2 h (kinetic experiments) or 4 h (permissiveness determination) post electroporation, to correct for transfection efficiency.

### HCV RNA quantification by Northern blotting

RNA preparation and Northern blotting were performed according to established procedures [22]. In essence, total cellular RNA was isolated from guanidinium isothiocyanate lysates by a phenol/chloroform based single-step protocol and denatured in glyoxal. Samples were analyzed by denaturing agarose gel electrophoresis and Northern hybridization. For strand specific detection of HCV RNA, radioactively labeled riboprobes encompassing nucleotides 6273 to 9678 of the JFH1 sequence were generated by T7- (minus-strand detection) or T3-polymerase (plus-strand detection) mediated *in vitro* transcription of plasmid pBSK-JFH1/6273-3′ [34]. Signals were recorded by phosphorimaging using a Molecular Imager FX scanner (BioRad, Munich, Germany) and quantified using the QuantityOne software (BioRad). To determine absolute molecule numbers, signals were quantified using serial dilutions of highly purified plus- and minus-strand *in vitro* transcripts of known quantity, which were loaded onto the same gel. Cross-hybridization of minus-strand probes with the plus-strand standard was observed to a low extent and corrected for.

### Microarray data

Permissiveness of eight Huh-7 derived cell-lines was assessed using a standard luciferase replication assay as described above. Total cellular RNA of untransfected cells was then isolated by Trizol extraction according to the manufacturer's protocol (Invitrogen, Karlsruhe, Germany), and gene expression was measured using the Affymetrix Human Genome U133 Plus 2.0 platform. Data were normalized in R/Bioconductor using RMA normalization. Genes were filtered using the variance-based (IQR) filter in nsFilter, and log2 fold-changes between high and low permissive cells were computed. We then fitted a linear model to the data, predicting replication efficiency in the eight cell lines from the corresponding gene expression values. ANOVA was used to assess statistical significance of individual genes. Hit selection was done using a relatively low threshold of 0.2 on the p-value and a log fold-change of at least 0.3, corresponding to a change in expression of approximately 25%. Resulting genes were intersected with published RNAi screening [46], [47], [48], [49] and virus-host protein interaction [45] data as described, yielding a list of 17 host factors that are differentially expressed between the high and low permissive cells, that correlate with replication permissiveness in the eight cell lines used, and that have previously been shown to be associated with HCV infection or replication. Genes were then mapped to pathways and annotated further using DAVID version 6.7 [74], [75] and IPA (Ingenuity Systems, www.ingenuity.com).

### Mathematical model

We developed a mathematical model using ordinary differential equations based on mass action kinetics. The model is subdivided into two compartments: 1) initial RNA processing, translation into the polyprotein and polyprotein processing (cleavage) occur in the cytoplasm, and 2) viral genome replication takes place inside of the replication compartment. A graphical summary of the model is shown in Figure 2C. The following set of equations was used to describe the processes in the two compartments:

Cytoplasm

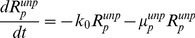
(1)

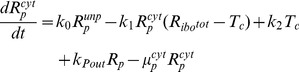
(2)

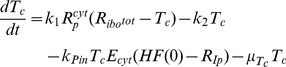
(3)

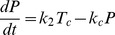
(4)

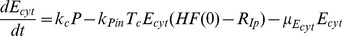
(5)

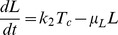
(6)Here, *R_p_^unp^* (eq. 1) represents the number of plus-strand RNA molecules entering the cell upon transfection. This transfected RNA is processed into translation competent *R_p_^cyt^* (eq. 2) at rate *k_0_*, describing, for example, transport and structural re-folding processes. The processed plus-strand RNA *R_p_^cyt^* interacts with ribosomes *R_ibo_* at a constant rate *k_1_* to form translation complexes *T_c_* (eq. 3), which degrade at rate *μ_Tc_*. Ribosomes are recovered when translation complexes *T_c_* degrade with rate *μ_Tc_*. Note that, as the total number of ribosomes in the cell (*R_ibo_^tot^*) is assumed constant, the number of ribosomes available for translation is given by *R_ibo_^tot^* – *T_C_*, and it is not necessary to introduce a separate equation for ribosomes. Unprocessed and processed RNAs *R_p_^unp^* and *R_p_^cyt^* degrade with rate constants *μ_p_^unp^* and *μ_p_^cyt^*, respectively (eq. 1 an 2). For simplicity, we assume that 10 ribosomes simultaneously translate the same HCV RNA [76], therefore, *R_ibo_^tot^* represents complexes consisting of 10 ribosomes. Viral polyprotein *P* is formed from *T_c_* at an effective rate *k_2_* (eq. 4). When the translation of polyprotein is complete, the translation complex dissociates into plus-strand RNA and ribosomes at rate *k_2_*. Newly produced polyprotein is cleaved with rate *k_c_* into the mature viral nonstructural (NS) proteins *E^cyt^* (eq. 5). NS proteins degrade at rate *μ_E_^cyt^*. Eventually, plus-strand RNA and NS proteins, most notably the polymerase NS5B, interact in *cis* and together with NS proteins in *trans* (*E^cyt^*) as well as a cellular factor *HF* to form a replication complex within the induced vesicular membrane structure. This *cis* interaction of *R_p_^cyt^* and translated NS proteins is realized in the model by requiring active translation complexes *T_c_* instead of free *R_p_^cyt^* for the formation of replication complexes. The host factor *HF* catalyzes the formation of *R_Ip_*, at the rate *k_Pin_*. Once *R_Ip_* is formed, ribosomes are freed again at rate *k_Pin_*. This leads to the ternary reaction *T_C_+E_Cyt_+HF→R_Ip_+R_Ibo_*, simultaneously describing formation of the replication compartments and initiation of minus strand RNA synthesis, compare also supplementary text S1 and supplementary figure S6. In turn, *HF* is freed again when *R_Ip_* degrades or upon completion of minus strand synthesis. As the total number of host factor molecules in the cell is assumed constant, we can replace *HF* by *HF(0) – RIp*, where *HF(0)* is the total number of HF molecules in the cell. Lastly, since we use a luciferase readout to measure polyprotein concentration, we furthermore include a luciferase marker *L* in the model, which is produced at the same rate as the polyprotein (*k_2_*), however does not require further processing and degrades with rate *μ_L_* (eq. 6).

Replication compartment

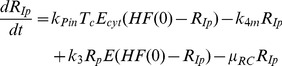
(7)

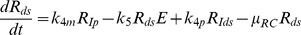
(8)

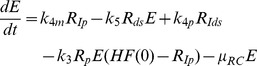
(9)

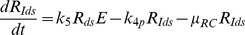
(10)

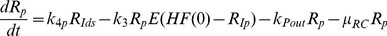
(11)
*R_Ip_* is the minus-strand RNA initiation complex (eq. 7), which contains a plus-strand RNA serving as template for the synthesis of minus-strand RNA. Minus strand RNA is synthesized from *R_Ip_* at rate *k_4m_*, yielding double stranded RNA *R_ds_* (eq. 8). We assume minus-strand RNA to be always bound to its complementary plus-strand in a double-stranded replicative intermediate. When the production of minus-strand RNA is complete, *R_Ip_* dissociates into *R_ds_*, *HF* and viral NS protein *E* (eq. 9). Next, *R_ds_* interacts again with *E* to form a plus-strand RNA initiation complex, *R_Ids_* (eq. 10), to initiate the synthesis of new plus-strands, *R_p_*, with a constant rate *k_4p_*, and dissociates into *R_ds_* and *E*. Newly synthesized plus-strand RNA, *R_p_* (eq. 11), then leaves the replication compartment at rate *k_Pout_* to participate in translation, or interacts with the polymerase *E* and host factor *HF* to again form the minus-strand RNA initiation complex *R_Ip_* at rate *k_3_*. For simplicity, we assume that the RNA *R_Ip_*, *R_ds_*, *R_Ids_* and *R_p_*, and proteins *E* all degrade with rate *μ_RC_*.

### Model parameters and parameter estimation

Reaction rates in the model were taken from literature as far as known, or estimated by fitting the model to the experimental data. Following Dahari et al [24], we used a value of *k_2_* = 100 polyproteins per hour per polysome for protein translation. RNA replication was assumed to occur at a rate of *k_4m_* = *k_4p_* = 1.7 viral RNA molecules per hour per replication complex, assuming plus- and minus-strand synthesis to occur at the same rate [23], [77], [78]. Based on an estimated half-life of Luciferase of approximately 2 hours, we estimated the corresponding degradation rate to be *μ_L_* = 0.35 h^−1^ [79], [80]. We furthermore estimated the NS protein half-life in the cytoplasm to be around 12 hours, corresponding to a rate of *μ_E_^cyt^* = 0.06 h^−1^ [76], [81], [82]. We observed from model calibration that the optimization would yield values with *μ_Tc_*>*μ_p_^cyt^*, violating the expectation that RNA in translation complexes should be more stable than free RNA in the cytoplasm. We hence added the constraint *μ_Tc_*/*μ_p_^cyt^* = 0.5, enforcing a 2-fold higher stability of RNA that is actively translated. We furthermore observed a low sensitivity of model output with respect to parameters *k_1_*, *k_c_*, *k_3_* and *k_5_*, compare figure 5, and hence fixed these parameters based on manual model analysis, for details see supplementary text S1.

Estimation of the remaining 7 model parameters, 3 initial values and a scale factor to convert luciferase measurements into polyprotein molecule numbers was done using multiple shooting, as implemented in the PARFIT package [83], [84], [85]. We simultaneously minimized the least squares prediction error on the high and low permissive cells in log-concentration space, using all individual measurements in the objective function. An additional scaling factor was introduced in the optimization problem to convert luciferase measurements for the viral polyprotein to molecule numbers. All model species containing viral plus-strand RNA or minus-strand RNA, respectively, were added for comparison with the experimental data, yielding *R_p_^tot^ = R_p_^unp^+R_p_^cyt^+T_c_+R_Ip_+R_ds_+R_Ids_+R_p_* for the total plus-strand RNA and *R_M_^tot^ = R_ds_+R_Ids_* for the total negative strand RNA concentrations. Ratios of RNA as reported in literature were furthermore used to constrain the optimization [10]. As some species attain very low values, we compared results of the approximation using differential equations with a stochastic solver (supplementary figure S7). For details of the parameter estimation and objective function used see supplementary text S1. Obtained model parameters and confidence intervals are shown in table 1.

### Identifiability analysis

To test our model for structural identifiability, we performed a local identifiability analysis at obtained optimal parameter values using SensSB [86]. Results of this analysis are shown in Supplementary Figure S8. High correlation between two parameters means that a change in the model output caused by a change in one parameter can be compensated by an appropriate change in the other parameter. This then prevents the parameters from being uniquely identifiable despite the output being very sensitive to changes in individual parameters. Parameters for which values were known from literature or which were fixed were also included in this identifiability analysis, to assess their effect on results. These parameters are indicated in grey in the Figure; several of these parameters are highly correlated with other parameters, thus reiterating the importance of experimental measurements for them. Importantly, the identifiability analysis indicates that most of the parameters that had to be calibrated from data showed low correlation to other parameters only, indicating an overall satisfactory identifiability of the model and, in particular, no indication of structural non-identifiability in the model with correlation values close to ±1.

We furthermore calculated confidence intervals on estimated model parameters using the covariance matrix of the parameters, as described in supplementary text S1. Most of the kinetic reaction rates had reasonable standard errors and confidence bands, while larger uncertainties were observed for the initial values, compare table 1. This sloppiness is typical for models in systems biology [87], [88]. Based on our aim to develop a predictive model and not uniquely identify individual reaction rates, our assessment was that the model is sufficiently identifiable for our purpose.

### Sensitivity analysis

Global sensitivity analysis was performed using the extended Fourier Amplitude Sensitivity Test (eFAST) [39], [40]. This algorithm calculates the first and total-order sensitivity indices of each parameter, and assesses the statistical significance of these sensitivity indices by a method based on dummy parameters. For details, we refer to Saltelli et al [89]. In brief, for a given model *y = f(x)* with scalar y and input vector *x* = (*x_1_, …, x_n_*), the first order sensitivity index with respect to *x_i_* is the expected amount of variance that would be removed from the total output variance, if we knew the true value of *x_i_*, divided by the total unconditional variance:
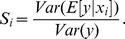

*S_i_* is a measure of the relative importance of the individual variable *x_i_* in driving the uncertainty in the output *y*. In contrast, the total sensitivity index with respect to a variable *x_i_* measures the residual output variance if only *x_i_* were left free to vary over its uncertainty range, and all other parameters were known:
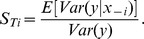

*S_Ti_* is a measure of how important a parameter is in determining the output variance, either singularly *or in combination* with other parameters. To assess the significance of obtained indices, eFast furthermore calculates the first and total order sensitivity index for a dummy parameter that is not part of the model. Indices that are not significantly larger than this dummy parameter index should not be considered different from zero [39].

Figures 6 and S4 show the resulting eFAST total order sensitivity indices of viral plus- and minus-strand RNA concentrations and viral polyprotein concentration with respect to the 16 model parameters and three initial values at two different time points, early in the viral lifecycle and after attainment of the steady state levels.

## Supporting Information

Figure S1 Fit of the original model by Dahari *et al.* [24] to our time-resolved measurements of positive strand RNA (blue), negative strand RNA (red) and polyprotein (black).
(EPS)

Figure S2 Quantitative assessment of alternative models to explain differences observed in HP and LP cells. Alternative models were set up to explain observed data, assuming that cells differ in (A) the initial RNA processing, (B) different numbers of ribosomes available for RNA translation, (C) different RNA degradation rates in the cytoplasm, (D) different polyprotein translation rates, (E) different rates of formation of the replication compartment, (F) different RNA synthesis rates inside the replication vesicles, (G) different RNA degradation rates inside the replication vesicles, and (H) different export rates of newly synthesized RNA into the cytoplasm. Models were fitted to the experimental data, and resulting *χ* ^2^ and Akaike Information Criterion (AIC) values compared. Line colors indicate polyprotein (black), plus-strand RNA (red) and minus-strand RNA (blue).
(EPS)

Figure S3 Comparison of activatory with consumed host factor (HF) model. The left plots show the model predictions in the high permissive cell line, the right plot shows the predictions for the low permissive cell line. Upper panels: activatory (enzymatic) HF model, lower panels: consumed HF model.
(EPS)

Figure S4 Global sensitivity analysis of the replication model. Sensitivity analysis was performed using the extended Fourier Amplitude Test (eFAST) at (A, C, E) 4 hours and (B, D, F) 72 hours. Shown are eFast total order sensitivity indices for (A, B) plus strand RNA, (C, D) minus strand RNA, and (E, F) viral protein. These total sensitivity indices account for first and higher order sensitivities involving each of the parameters indicated. The dashed horizontal lines are sensitivities of a negative control parameter that does not occur in any of the equations, and are thus a measure of background variability of the sensitivity estimation procedure. Sensitivities lower or equal to the dashed line should not be considered as significantly different from zero.
(EPS)

Figure S5 The figure shows attained steady state levels of positive strand RNA, for different values of the RNA degradation rate *μ_RC_* in the replication compartment. Note the transition at *μ_RC_* = 0.4, where a switch occurs from low-level persistent replication to complete clearance of the infection.
(EPS)

Figure S6 The figure replaces the ternary interaction *T_c_+E_cyt_+HF→R_Ip_+R_ibo_* by two binary reactions, assuming that *T_c_* and *E_cyt_* bind first, forming an intermediate complex *C* in a reversible reaction with rates *k_a_* (forward reaction) and *k_b_* (backward reaction), that then irreversibly reacts with *HF* to yield *R_Ip_* and *R_ibo_* with rate *k_c_*. The figure in panel (A) was obtained by fixing parameter *k_a_* to 3e-4, varying parameter *k_b_* between 1 and 200, and then optimizing parameter *k_c_* to fit the experimental data. The plot shows that increases in *k_b_* can be compensated by increases in *k_c_*, rendering the model practically non-identifiable. Panels (B) and (C) show the obtained fits to the data for *k_b_* = 10 and *k_b_* = 200, with associated values *k_c_* = 0.41 and *k_c_* = 7.64, respectively, and *χ* ^2^ values of 3.55 and 2.45, respectively. Blue line and points: (-) RNA, Red: (-) RNA, Black: Viral Polyprotein (Luciferase).
(EPS)

Figure S7 Ten different runs for each high and low permissive cells, using a stochastic solver (implicit tau method) to make simulations with our calibrated replication model. Individual runs show a very similar behavior to the deterministic ordinary differential equation model, indicating that stochastic effects to not play a major role in determining the overall dynamics of the model.
(EPS)

Figure S8 Correlation between model parameters from identifiability analysis using SensSB [86]. High correlation between two parameters means that a change in the model output caused by a change in one parameter can be compensated by an appropriate change in the other parameter. This then prevents the parameters from being uniquely identifiable despite the output being very sensitive to changes in individual parameters. Parameters for which values were known from Literature or which were fixed after identifiability analysis are indicated in grey in the Figure. The Figure shows that most parameters are identifiable at optimal values obtained from model fitting. Parameters *μ_E_^cyt^* and *μ_L_*, which are highly correlated, however, the value for *μ_E_^cyt^* is known from literature and is not calibrated using the data [76], [81], [82], rendering the second parameter *μ_L_* identifiable. Similarly, the high correlation seen between *R_ibo_^tot^* and *k_2_* as well as the high correlation between *HF_0_* and *k_4m_* are unproblematic, as parameters *k_2_* and *k_4m_* were set based on literature data [23], [24], [77], [78]. The correlation seen between *k_Pout_* und *μ_p_^cyt^*, is unproblematic, as an additional constraint *μ_p_^cyt^* = 2 *μ_Tc_* on *μ_p_^cyt^* is used in the parameter estimation.
(EPS)

Table S1 Differentially expressed genes between the eight cell lines analyzed. The first column is the gene name, the second column the corresponding Affymetrix ID. The logfc column is the logarithm of the fold expression change between the high and low permissive cells, whereas the p-value is computed from an analysis of variance of the full panel of all eight cell lines.
(XLS)

Table S2 Differentially expressed genes between the eight cell lines analyzed, showing the 355 genes with log-fold change >0.3 or <−0.3 between high and low permissive cells, and p-value<0.2 in correlation analysis with permissiveness over all 8 cell lines. The table gives log fold-changes for all 8 cell lines, as well as p-value of correlation for all genes.
(XLS)

Table S3 Annotation of 355 differentially expressed genes correlating with permissiveness to cellular function categories. Analysis was done using IP (Ingenuity Systems, www.ingenuity.com). The functional analysis identified the biological functions that were most significant to the data set. Molecules from the dataset that met the p-value<0.2 and log fold-change >0.3 or <−0.3 criteria were associated with the biological functions in the Ingenuity Knowledge Base. Right-tailed Fisher's exact test was used to calculate a p-value determining the probability that each biological function assigned to the data set is due to chance alone. Shown are annotations for category and cellular function, together with p-value, number and names of respective molecules.
(XLS)

Table S4 Annotation of 255 differentially expressed genes correlating with permissiveness to canonical pathways. Analysis was done using IP (Ingenuity Systems, www.ingenuity.com). Canonical pathway analysis identified the pathways from the IPA library of canonical pathways that were most significant to the data set. Molecules from the data set that met the p-value<0.2 and log fold-change >0.3 or <−0.3 criteria and were associated with a canonical pathway in the Ingenuity Knowledge Base were considered for the analysis. The significance of the association between the data set and the canonical pathway was measured in 2 ways: 1) A ratio of the number of molecules from the data set that map to the pathway divided by the total number of molecules that map to the canonical pathway is displayed. 2) Fisher's exact test was used to calculate a p-value determining the probability that the association between the genes in the dataset and the canonical pathway is explained by chance alone.
(XLS)

Table S5 Parameter estimates for the HCV replication model (consumed *HF*).
(PDF)

Text S1 The supplementary text contains additional information on gene expression analysis, model development and parameter estimation, and model analysis.
(PDF)
