# Supplementary material for: Replication Vesicles are Load- and Choke-Points in the Hepatitis C Virus Lifecycle
Source: PLoS Pathog. 2013 Aug 22;9(8):e1003561. doi: 10.1371/journal.ppat.1003561 (PMC3749965; doi:10.1371/journal.ppat.1003561)
Supplement: Table S5 — Parameter estimates for the HCV replication model (consumed HF). (PDF) [file ppat.1003561.s013.pdf]

Supplementary Table S5. Parameter estimates for the HCV replication model (consumed HF).

Rate constant	Definition	Rate constant	90% confidence interval	Reference
k_0	Processing rate of transfected positive-strand RNA	0.0094 h^{-1}	(2.7e-3, 3.2e-2)	
k_1	Formation rate of translation complex	1 h^{-1} $molecule^{-1}$	Fixed after sensitivity / identifiability analysis	
k_2	Polyprotein translation rate	100 h^{-1}	Experimentally observed	(Dahari et al, 2007)
k_c	Polyprotein cleavage rate	1 h^{-1}	Fixed after sensitivity / identifiability analysis	
k_{Pin}	Formation rate of the plus-strand replicative intermediate complex	7.0e-8 h^{-1} $molecule^{-2}$	(7.14e-9, 6.9e-7)	
k_{Pout}	Transport rate of nascent plus-strand RNA into cytoplasm	0.36 h^{-1}	(0.23, 0.57)	
k_3	Formation rate of the plus-strand replicative intermediate complex from within the RC	10^{-4} h^{-1} $molecule^{-1}$	Fixed after sensitivity / identifiability analysis	
k_{4m}	Minus-strand RNA synthesis rate	1.7 h^{-1}	Experimentally observed	(Lohmann et al, 1999; Ma et al, 2005; Oh et al, 1999)
k_{4p}	Plus-strand RNA synthesis rate			
k_5	Formation rate of the minus-strand replicative intermediate complex	10 h^{-1} $molecule^{-1}$	Fixed after sensitivity / identifiability analysis	
μ_P^{unp}	Degradation rate of unprocessed plus-strand RNA	0.77 h^{-1}	(0.51, 1.1)	
μ_P^{cyt}	Degradation rate of processed plus-strand RNA	0.57 h^{-1}	(0.32, 1.0)	
μ_{Tc}	Degradation rate of translation complex	0.29 h^{-1}	(0.16, 0.51)	
μ_E^{cyt}	Degradation rate of NS5B protein	0.06 h^{-1}	Experimentally observed	(Pause et al, 2003; Pietschmann et al, 2001; Wang et al, 2003)
μ_{RC}	Degradation rate of RNA and protein in Replication Compartment	0.0021 h^{-1}	(2.2e-10 1.9e4)	
μ_L	Degradation rate of luciferase	0.35 h^{-1}	Experimentally observed	(Leclerc et al, 2000; Thompson et al, 1991)
$HF_{high}(0)$	Initial values for activated host factor in high permissive	650 molecules	(200, 2200)	

	cells			
$HF_{low}(0)$	Initial values for activated host factor in low permissive cells	87 molecules	(23, 330)	
$R_{ibo}(0)$	Total ribosome complexes	1900 molecules	(360, 9900)	
f_{Scale}	Scaling factor for Luciferase polyprotein marker	4300	(1600, 12000)	
