# Supplementary material for: Replication Vesicles are Load- and Choke-Points in the Hepatitis C Virus Lifecycle
Source: PLoS Pathog. 2013 Aug 22;9(8):e1003561. doi: 10.1371/journal.ppat.1003561 (PMC3749965; doi:10.1371/journal.ppat.1003561)
Supplement: Text S1 — The supplementary text contains additional information on gene expression analysis, model development and parameter estimation, and model analysis. (PDF) [file ppat.1003561.s014.pdf]

Supplementary Text

Replication Vesicles are Load- and Choke-Points in the Hepatitis C Virus Lifecycle

M. Binder, N. Sulaimanov, D. Clausznitzer, M. Schulze, C. M. Hüber, S. M. Lenz, J. P. Schlöder, M. Trippler, R. Bartenschlager, V. Lohmann and L. Kaderali

1. Gene Expression Analysis

Permissiveness of eight Huh-7 derived cell-lines was assessed using a standard luciferase replication assay. Total cellular RNA of untransfected cells was then isolated by Trizol extraction according to the manufacturer's protocol (Invitrogen, Karlsruhe, Germany), and gene expression was measured using the Affymetrix Human Genome U133 Plus 2.0 platform. Data were normalized in R/Bioconductor using RMA normalization. Genes were filtered using the variance-based filter (IQR) in nsFilter, and log2 fold-changes between high and low permissive cells computed. We then fitted a linear model to the data, predicting replication efficiency in the eight cell lines from the corresponding gene expression values. ANOVA was used to assess statistical significance of individual genes. Hit selection was done using a relatively low threshold of 0.2 on the p-value and a log fold-change of at least 0.3, corresponding to a change in expression of approximately 25%. Resulting genes were intersected with published RNAi screening data and virus-host protein interaction data as described in the methods section of the main manuscript, yielding a list of 17 host factors that are differentially expressed between the high and low permissive cells, that correlate with replication permissiveness in the eight cell lines used, and that have previously been shown to be associated with HCV infection or replication. Genes were then mapped to pathways and Gene Ontology Annotations analyzed using DAVID version 6.7 [1,2] and IPA (Ingenuity® Systems, www.ingenuity.com).

The full list of genes on the microarrays together with the fold-changes and p-values are shown in supplementary table S1. Supplementary table S2 shows the 355 genes correlated with permissiveness in the 8 cell lines, with at least 25% change of expression between high and low permissive cell lines and p-value <0.2 in analysis of correlation between permissiveness and expression. Supplementary tables S3 and S4 contain functional and pathway annotation for the respective genes generated using IPA (Ingenuity® Systems, www.ingenuity.com)

2. Model Calibration

The final replication model comprises 13 molecular species (two of them implicit, without separate equations), and is parameterized by 16 parameters corresponding to reaction rates, as well as three non-zero initial values and one scaling parameter. As the total amount of Ribosomes in the cell (R_{ibo}^{tot}) is assumed constant and equal to the sum of free Ribosomes and Ribosomes bound in translation complexes T_c, we do not need to include a separate equation for the free ribosomes (R_{ibo}), but can rather calculate them implicitly as $R_{ibo} = R_{ibo}^{tot} - T_c$. Similarly, the total amount of Host Factor $HF^{tot}=HF(0)$ is assumed constant, and is equal to $HF(0) = HF + R_{lp}$. Reaction rates in the model were taken from literature as far as known, or estimated by fitting the model to the experimental data. For simplicity, we assumed that all RNA species in the RC degrade at the same rate μ_{RC}. Following [3], we used a value of k_2=100 polyproteins per hour per polysome for protein translation. RNA replication was assumed to occur at a rate of $k_{4m} = k_{4p}$ = 1.7 viral RNA molecules per hour per replication complex, assuming plus- and minus-strand synthesis to occur at the same rate [4,5,6]. Based on an

estimated half-life of Luciferase of approximately 2 hours, we estimated the corresponding degradation rate to be μ_L = 0.35 h^{-1} [7,8]. We furthermore estimated the NS protein half-life in the cytoplasm to be around 12 hours, corresponding to a rate of μ_E^{cyt} = 0.06 h^{-1} [9,10,11]. We observed from model calibration that the optimization would yield values with $\mu_{Tc} > \mu_p^{cyt}$, violating the expectation that RNA in translation complexes should be more stable than free RNA in the cytoplasm. We hence added the constraint $\mu_{Tc} / \mu_p^{cyt} = 0.5$, enforcing a 2-fold higher stability of RNA that is actively translated. We furthermore observed a low sensitivity of model output with respect to parameters k_1, k_c, k_3 and k_5, compare figure 5 in the main manuscript and supplementary figure S5, and hence estimated these parameters based on manual model analysis. The effect of these equality constraints was thoroughly evaluated to assess the impact of these assumptions, as described below.

Formulation of the Parameter Estimation Problem

For the estimation of the remaining 7 parameters, three initial values and scale factor, we consider the following nonlinear, multi-experiment least-squares optimization problem, which is subject to equality and inequality constraints:

$$\begin{aligned}
&\min_{y^1(\bullet),\dots,y^{m_3}(\bullet),p} \sum_{r=1}^{m_3}\sum_{j=1}^{m_2}\sum_{i=1}^{m_1}\left(w_{ij}^r\left(\eta_{ij}^r - h_j(t_i, y^r(t_i), p)\right)\right)^2 \\
&\textit{subject to } F_{eq,k}(\{y^r(t_i)\}_{i=1,\dots,m_1}^{r=1,\dots,m_3}, p) = 0, \qquad k = 1,\dots,n_{eq} \\
&\qquad F_{in,l}(\{y^r(t_i)\}_{i=1,\dots,m_1}^{r=1,\dots,m_3}, p) \geq 0, \qquad l = 1,\dots,n_{in} \\
&\qquad \dot{y}^r(t) = f(t, y^r(t), p) \quad \textit{for} \quad t \in [t_0^r, t_f^r]
\end{aligned} \tag{1}$$

Herein, $y^r(t)$ is the state vector of the differential equation system at time t in experiment r, which in our model is given by the concentrations of the 11 species (R_p^{unp}, R_p^{cyt}, T_c, P, E_{cyt}, R_{Ip}, R_{ds}, E, R_{Ids}, R_p and L) at time t in experiment r. The vector p represents the unknown parameters to be estimated, including the scaling factor for the Luciferase measurements, and the initial values for the host factor in the two cell lines, as well as the total number R_{ibo}^{tot} of Ribosomes available for translation. The right hand side function f of the differential equation system is given by the model equations in the cytoplasm and replication compartment:

Cytoplasm:

$$(1) \quad \frac{dR_p^{unp}}{dt} = -k_0 R_p^{unp} - \mu_p^{unp} R_p^{unp}$$

$$(2) \quad \frac{dR_p^{cyt}}{dt} = k_0 R_p^{unp} - k_1 R_p^{cyt}(R_{ibo^{tot}} - T_c) + k_2 T_c + k_{Pout} R_p - \mu_p^{cyt} R_p^{cyt}$$

$$(3) \quad \frac{dT_c}{dt} = k_1 R_p^{cyt}(R_{ibo^{tot}} - T_c) - k_2 T_c - k_{Pin} T_c E_{cyt}(HF(0) - R_{Ip}) - \mu_{Tc} T_c$$

$$(4) \quad \frac{dP}{dt} = k_2 T_c - k_c P$$

$$(5) \quad \frac{dE_{cyt}}{dt} = k_c P - k_{Pin} T_c E_{cyt}(HF(0) - R_{Ip}) - \mu_{E_{cyt}} E_{cyt}$$

$$(6) \quad \frac{dL}{dt} = k_2 T_c - \mu_L L$$

Replication Compartment:

$$(7) \qquad \frac{dR_{Ip}}{dt} = k_{Pin} T_c E_{cyt}(HF(0) - R_{Ip}) - k_{4m} R_{Ip} + k_3 R_p E(HF(0) - R_{Ip}) - \mu_{RC} R_{Ip}$$

$$(8) \qquad \frac{dR_{ds}}{dt} = k_{4m} R_{Ip} - k_5 R_{ds} E + k_{4p} R_{Ids} - \mu_{RC} R_{ds}$$

$$(9) \qquad \frac{dE}{dt} = k_{4m} R_{Ip} - k_5 R_{ds} E + k_{4p} R_{Ids} - k_3 R_p E(HF(0) - R_{Ip}) - \mu_{RC} E$$

$$(10) \qquad \frac{dR_{Ids}}{dt} = k_5 R_{ds} E - k_{4p} R_{Ids} - \mu_{RC} R_{Ids}$$

$$(11) \qquad \frac{dR_p}{dt} = k_{4p} R_{Ids} - k_3 R_p E(HF(0) - R_{Ip}) - k_{Pout} R_p - \mu_{RC} R_p$$

The number m_1 in the optimization problem (1) is the number of different time points available. The number m_3 denotes the number of different experiments, in our case $m_3=2$, because we have measurements for high and low permissive cells. The number m_2 denotes the number of different measurement types, which in our case is $m_2=6$, because we have measurements for plus-strand RNA, minus-strand RNA, and Luciferase, and three steady-state ratios at $t=72$, which we include in the objective function [12]: (1) A ratio of 10:1 of plus to minus strand RNA; (2) a ratio of 6:1 of plus to minus strand RNA in the replication vesicles; (3) a ratio of 1:1 of plus strand RNA in the cytoplasm to plus strand RNA in the replication compartment.

Further, η_{ij}^r denotes the value of the measurement of type j taken at time t_i in experiment r. For each measurement type, there is a function h_j, called measurement function or measurement model, which simulates the outcome of a measurement of this type at time t_i. For plus-strand RNA measurements, the measurement function is given by $\log_{10}(R_p^{cyt} + T_c + R_p + R_{ds} + R_{Ip} + R_{Ids} + R_p^{unp})$, for minus-strand RNA measurements, the measurement function is given by $\log_{10}(R_{ds} + R_{Ids})$, and for Luciferase measurements, the measurement function is given by $\log_{10}(L) - \log_{10}(f_{Scale})$, where f_{Scale} is the scaling factor for the Luciferase measurements. The measurement functions for the ratios are

$$(R_p^{cyt} + T_c + R_p + R_{ds} + R_{Ip} + R_{Ids} + R_p^{unp}) \,/\, (R_{ds} + R_{Ids})$$

for total plus-strand RNA to total minus-strand RNA,

$$(R_p + R_{ds} + R_{Ip} + R_{Ids}) \,/\, (R_{ds} + R_{Ids})$$

for plus-strand RNA in VMS to minus-strand RNA in the RC, and

$$(R_p^{cyt} + T_c + R_p^{unp}) \,/\, (R_p + R_{ds} + R_{Ip} + R_{Ids})$$

for plus-strand RNA in cytoplasm to plus-strand RNA in the RC.

Finally, the factor w_{ij}^r is equal to one whenever a measurement of type j has been taken at time t_i in experiment r, and zero otherwise.

Multiple Shooting Parameterization

The optimization problem given above is an infinite-dimensional optimization problem, because the functions $y^r(\cdot)$ occur as optimization variables, which have to fulfill the differential equation (an infinite-dimensional equality constraint). In order to reduce the optimization problem to finite dimension, we use the multiple shooting method [13,14,15], which has successfully been applied to real world parameter estimation problems in many different application areas like chemical engineering (e.g. the denitrogenization of pyridine, see [13], biophysics, e.g. the photosynthesis process [16], and civil space flight, e.g. satellite orbit determination [17]).

Using multiple shooting, the finite dimensional vector of optimization variables is given by $(s_0^1,\ldots,s_M^1,\ldots,s_0^{m_3},\ldots,s_M^{m_3},p)$, where s_k^r are the state vectors at the so-called multiple shooting nodes $\tau_k^r, k=0,\ldots,M$, where $\tau_0^r = t_0^r$ and $\tau_M^r = t_f^r$ (for simplicity it is assumed that the same number of multiple shooting nodes is used in all experiments). Each evaluation of the state vector $y^r(t_i)$ is computed by solving an initial value problem on the corresponding multiple shooting interval, i.e. if $t_i \in [\tau_k^r, \tau_{k+1}^r)$ then $y(t_i; s_k^r, p)$ is the solution of the initial value problem

$$\dot{y}(t) = f(t, y(t), p)$$
$$y(\tau_k^r) = s_k^r$$

evaluated at t_i.

The notation $y(t_i;s_k^r,p)$ indicates that the initial value problem solution depends on the initial value and the parameters. In order to ensure that $y^r(t)$ is continuous in the solution of the optimization problem for all experiments r, additional equality constraints – so-called matching conditions – are imposed, which require that the state variables s_{k+1}^r are equal to the solution of the initial value problem on the preceding interval, i.e. $s_{k+1}^r - y(\tau_{k+1}^r;s_k^r,p) = 0, \quad for \quad k=0,\ldots,M-1$. Thus, we arrive at the following finite-dimensional, nonlinear least-squares optimization problem:

$$\min_{s_0^1,\ldots,s_M^1,\ldots,s_0^{m3},\ldots,s_M^{m3},p} \sum_{r=1}^{m_3}\sum_{j=1}^{m_2}\sum_{i=1}^{m_1}\left(w_{ij}^r\left(\eta_{ij}^r - h_j(t_i,y^r(t_i),p)\right)\right)^2$$

$$subject\ to\ F_{eq,k}(\{y^r(t_i)\}_{i=1,\ldots,m_1}^{r=1,\ldots,m_3},p)=0, \qquad k=1,\ldots,n_{eq}$$

$$F_{in,l}(\{y^r(t_i)\}_{i=1,\ldots,m_1}^{r=1,\ldots,m_3},p)\geq 0, \qquad l=1,\ldots,n_{in}$$

$$s_{k+1}^r - y(\tau_{k+1}^r;s_k^r,p) \quad = \quad 0, \quad for \quad k=0,\ldots,M-1$$

The well-known and commonly used single shooting method, where a finite-dimensional optimization problem is obtained by using only the initial value $s_0^r = y^r(t_0)$ in each experiment r as optimization variable, is derived from the above optimization problem by setting $M = 0$ and omitting the matching conditions. However, the usage of multiple shooting, i.e. the introduction of additional optimization variables and equality constraints, is known to have many theoretical and practical advantages, like improved convergence behavior through a suitable initialization of the augmented vector of optimization variables [17], decreased nonlinearity of the problem (even down to one-step convergence, see [15]) , and the possibility to estimate parameters in systems with unstable behavior, which cannot be treated using the single shooting method [14].
Collecting all components of F_{eq} and the matching conditions into a single vector, and using the definition $x=(s_0^1,\ldots,s_M^1,\ldots,s_0^{m_3},\ldots,s_M^{m_3},p)$ for the vector of optimization variables, the optimization problem can be written as

$$\min_x \|F_1(x)\|_2^2$$

$$subject\ to\ F_2(x)=0$$

$$F_3(x)\geq 0,$$

where the equality and inequality constraints have to be fulfilled component-wise. In our case, all inequality constraints are bounds to the parameter values and were treated by adding suitable smooth potential terms to the least squares objective function, which keep the parameters within biologically plausible ranges.

Generalized Gauss-Newton Method

The remaining equality-constrained nonlinear least squares function is solved using the Generalized Gauss-Newton method, i.e. starting from an initial guess x^0 for the optimization variables, we iterate $x^k \rightarrow x^{k+1} = x^k + \Delta x^k$, where the increment Δx^k is determined as the solution of a linearized constrained least-squares problem:

$$\min_{\Delta x^k} \left\| F_1(x^k) + J_1(x^k)\Delta x^k \right\|_2^2$$
$$\textit{subject to } F_2(x^k) + J_2(x^k)\Delta x^k = 0,$$

where

$$J_i(x^k) = \frac{\partial F_i(x)}{\partial x}\Big|_{x=x^k}$$

is the Jacobian matrix of the function $F_i(x)$.
Using the definitions

$$F(x^k) = \begin{pmatrix} F_1(x^k) \\ F_2(x^k) \end{pmatrix} \text{ and } J(x^k) = \begin{pmatrix} J_1(x^k) \\ J_2(x^k) \end{pmatrix},$$

we can write the solution of the linearized problem formally as

$$\Delta x^k = -J^+(x^k)\begin{pmatrix} F_1(x^k) \\ F_2(x^k) \end{pmatrix},$$

where $J^+(x^k)$ is the generalized inverse of $J(x^k)$, which is given by

$$J^+(x^k) = -\begin{pmatrix} I & 0 \end{pmatrix}\begin{pmatrix} J_1^T(x^k)J_1(x^k) & J_2^T(x^k) \\ J_2(x^k) & 0 \end{pmatrix}^{-1}\begin{pmatrix} J_1^T(x^k) & 0 \\ 0 & I \end{pmatrix},$$

where I denotes the identity matrix. For the practical numerical solution, Householder decompositions of the Jacobian matrices are used. By exploiting the block-diagonal structure in the Jacobian J_2, which arises from the matching conditions, the solution of linear problems is done very efficiently. In order to improve the convergence behavior of the Gauss-Newton method, we use damped iterations $x^k \rightarrow x^{k+1} = x^k + \lambda^k \Delta x^k$, where the damping factor $\lambda^k \in (0,1]$ is obtained from the restrictive monotonicity test [18]. Ill-conditioned parameter combinations, which correspond to small diagonal elements in the triangular matrices of the Householder decomposition of J_1, are regularized.

Computation of Initial Value Problem Solutions and Derivatives

As described in the section "Multiple Shooting Parameterization", the evaluation of the state vectors and $y(t_i;s_k,p)$ and $y(\tau_{k+1};s_k,p)$(for the matching conditions) requires the solution of initial values problems on the multiple shooting intervals. We compute these initial value problem solutions using a variable stepsize extrapolation method based on the semi-implicit midpoint rule [19]. Derivatives of initial value problem solutions with respect to initial values and parameters, $\frac{\partial y(t\ ;s_k,p)}{\partial(s_k,p)}$, which are needed for the derivative-based optimization method, are computed using Internal Numerical Differentiation. This method allows a very reliable and efficient computation of the derivatives [13,14].

Statistical Analysis

In the solution x^* of the parameter estimation problem we perform a statistical analysis based on the covariance matrix C, which can be computed from the generalized inverse by

$$C = \beta_c^2 J^+(x^k)\begin{pmatrix} I & 0 \\ 0 & 0 \end{pmatrix}(J^+(x^k))^T \cdot$$

Herein, I denotes the identity matrix of the dimension of the number of least-squares conditions and β_c^2 denotes the so-called common factor, which is computed by

$$\beta_c^2 = \frac{\left\| F_1(x^*) \right\|_2^2}{l_2},$$

where l_2 denotes the number of degrees of freedoms, i.e. the number of optimization variables minus the number of equality constraints. Estimated standard errors for all optimization variables are given by the square roots of the diagonal elements of the covariance matrix. A confidence interval for significance level α for the optimization variable x_i can be obtained by $x_i = [x_i + \delta x_i, x_i - \delta x_i]$, where $\delta x_i = \sqrt{C_{ii}}\sqrt{l_1 F_{1-\alpha}(l_1, l_2)}$, where l_1 is the number of least-squares conditions minus l_2 and $F_{1-\alpha}(l_1, l_2)$ is the quantile of the F distribution for error probability $1-\alpha$.
Further, by applying a singular value decomposition of the Jacobian J_1 on the null-space of the equality constraints $J_{1\perp}(x^*) = USV^T$, it is also possible to see the correspondence of small singular values (diagonal elements of S) to the associated parameter combinations (rows of the matrix V^T).

Software

The above described numerical methods for the solution of nonlinear constrained least squares problems and the statistical analyses are implemented in the software package PARFIT, which was used for the parameter estimation of our model. The initial value problem solutions and their derivatives are computed using the solver METANB, which is incorporated in PARFIT.
Supplementary Table S5 shows the obtained estimates for the model parameters, together with 90% confidence intervals.

3. Statistical Model Analysis and Analysis of Model Constraints

We performed an analysis of structural model identifiability, using SensSB [20]. SensSB is a Matlab toolbox that integrates a variety of local and global sensitivity and identifiability analysis methods. We performed a local sensitivity analysis of the full model using SensSB. Results of this analysis are shown in Supplementary Figure S8. High correlation between two parameters means that a change in the model output caused by a change in one parameter can be compensated by an appropriate change in the other parameter.

We furthermore performed a sensitivity analysis of obtained model parameters using the extended Fourier Amplitude Sensitivity Test (eFAST) [21,22], shown in Figure 5 in the main manuscript and supplementary figure S4. Based on these analyses, we observed a low sensitivity of model output with respect to parameters k_1, k_c, k_3 and k_5 for all measured species (+RNA, -RNA and Protein) and hence fixed these parameters manually to plausible values, as given in Supplementary Table S5. We observed from model calibration that the optimization would yield values with $\mu_{Tc} > \mu_p^{cyt}$, violating the expectation that RNA in translation complexes should be more stable than free RNA in the cytoplasm.

We hence added the constraint μ_{Tc} / μ_p^{cyt} = 0.5, enforcing a 2-fold higher stability of RNA that is actively translated. We then examined the effect of these additional constraints in the parameter estimation individually. This is done by removing or changing each constraint individually, recalibrating the full model, and then discussing the changes seen both in the objective function, the obtained fits, as well as changes in obtained model parameters.

Effect of constraint on k_1, the formation rate of the translation complex T_c

The effect of the constraint on k_1 is investigated by fixing this parameter to the alternative values 0.5, 10.0, and 20.0.

A small decrease in the objective function can be achieved by changing the constraint to k_1 = 0.5, i.e. below the lower bound (decrease of 1.5%, LSQSUM = 10.579). Recalibration of the full model then shows that the largest changes in parameter values are obtained for k_{Pin} (increases to 2.0e-5), R_{Ibo}^{tot} (decreases to 390), and the scale factor (decreases to 1400). These changes are well within the given confidence intervals.

For larger values of k_1, the objective function increases slightly (1.6%, LSQSUM = 10.926, for k_1=20). The parameter values change again only within the confidence intervals.

These results indicate, that essentially changes in the formation rate k_1 of the translation complex T_c can be balanced by changing the available number of ribosomes and by changing the import rate of the translation complexes into the replication compartment, then giving comparable results. This parameter is difficult to address experimentally, we hence decided to fix it to a plausible value of 1/(h*molecule).

Effect of the constraint on k_c, the polyprotein cleavage rate

The effect of the constraint on k_c is investigated by fixing it alternatively to the values of 0.1, 0.5, 2.0 and 5.0. The least-squares sum changes only negligibly (less than 1%) for k_c = 0.5, 1, and 2. The values of the other parameters change by less than 20%. For k_c = 0.1, the least-squares sum increases to 11.109 (+3.4%). k_{Pin} increases to 1.9e-5, k_0 increases to 5.4e-3, and R_{Ibo}^{tot} increases to 890. All these values are within the confidence intervals. This indicates that this parameter is ill conditioned, and cannot be estimated accurately from the data given.

When considering the structure of the model, this result is not surprising, and due to the linear cascade of processes in the cytoplasm (protein translation followed by cleavage followed by import into the replication compartment). Within this cascade, k_c is not rate limiting, hence changes in the cleavage rate hardly affect results. Based on these considerations, we decided to fix k_c to a value of 1/h.

Effect of the constraint on k_3, the formation rate of the plus strand replication complex within the replication compartment

The effect of the constraint on k_3 is investigated by fixing it on the values 1e-3 and 1e-5, respectively. The least-squares sum decreases slightly for k_3 = 1e-5 to 10.702 (-0.4%), and increases notably to 11.0 (+2.2%) for k_3 = 1e-3. Other parameter values are almost unchanged (less than 10%). The nominal value 1e-4 seems to be an upper bound that is consistent with the measurement data (i.e. lower values are acceptable and lead to a slightly decreased objective function value, whereas higher values lead to a significant increase in the objective function value).

Effect of the constraint on k_5, the formation rate of the minus strand replication complex

The effect of the constraint on k_5 is investigated by fixing this parameter to the values 1, 5, 100, and 500. For both the larger and the smaller values, the least squares sum changes only marginally (up to

1.3% for k_5 = 500). The largest deviations for the parameter values are again observed for k_{Pin} (varies between 5e-6 and 2.4e-5), scale factor (between 1600 and 2900), and R_{Ibo}^{tot} (between 420 and 1000). Again, these variations are within the confidence intervals.

Effect of the constraint $\mu_{Tc} = 0.5*\mu_p^{cyt}$

Based on the biologically motivated assumption that the degradation rate of actively translated RNA in complexes should be less than that of free RNA in the cytoplasm, we included the constraint $\mu_{Tc} = 0.5*\mu_p^{cyt}$ in the model. The effect of this constraint is investigated by simply removing it from the optimization problem.

Without the constraint, the objective function decreases to 10.195 (-5.2%). As a result, μ_{Tc} = 0.29 and μ_p^{cyt} = 0.23 < μ_{Tc} is obtained. The largest change in any of the other parameters is observed for k_{Pin} (1.4e-5, +50%), whereas all other parameters change by less than 20%.

4. Effect of Host Factor – Consumed versus Enzymatic

One of the assumptions made in our model is the participation of a crucial host factor involved in the formation of the replication compartment / the structural rearrangements occurring in the rough endoplasmatic reticulum. While the nature of the host factor is not specified in more detail in our model, there are two different possibilities how this host factor might be involved: (1) as an active component of the structural rearrangements, being consumed in the process [consumed HF model], or (2) as an enzyme, that catalyzes the reactions, but is released afterwards and is not consumed [activatory HF model].

We evaluated and compared both of these model alternatives. The dynamics and parameters for both models are very comparable, as shown in Supplementary Figure S3. Table 1 on the main manuscript shows the corresponding parameters estimated for the activatory model, supplementary table S5 shows the parameters for the consumed model.

5. Model Selection

We developed our model based on a model published by Dahari and coauthors [3]. This model was developed based on steady state measurements of viral RNA and polyprotein, and we determined that the model was not able to explain the initial replication dynamics as observed in our data. Furthermore, the model cannot explain the differences between high and low permissive cells.

In order to compare possible hypotheses about what differences in the considered Huh cell lines could give rise to the observed dynamics, we implemented different assumptions about differences in high- and low permissive cell lines in Dahari's model, and re-fitted the full model simultaneously to the hp and lp cells, requiring all parameters to be identical between the two cell lines, except for the parameters relating to the hypothesis under consideration. Figure S2 shows the resulting time courses, chi-squared values and AIC values obtained.

6. Ternary interactions for host factor model

A central assumption made in our model is that translation complexes T_c, host factor HF and cytoplasmic proteins E_{cyt} act in cis for the initiation of minus strand RNA synthesis. This is implemented in our model through ternary reactions $T_c + E_{cyt} + HF \rightarrow R_{Ip} + R_{ibo}$. This is a model assumption that we made to avoid introducing additional parameters in the model. We tested the consequences of these assumptions by splitting the ternary interactions into two sets of binary reactions with intermediate complex C, as follows:

(1) $T_c + E_{cyt} \underset{k_b}{\overset{k_a}{\rightleftarrows}} C$

(2) $C + HF \xrightarrow{k_c} R_{Ip} + R_{ibo}$

We then re-calibrated the three additional model-parameters using an interior-point method. By iterating this from different starting points, we observed that parameter k_a consistently obtained values around 3e-4, whereas parameters k_b and k_c seemed correlated, i.e. increasing values of k_c could be compensated by increasing k_b and vice versa, without significantly affecting the quality of the obtained fit. To study this further, we fixed k_b to different values between 1 and 200, and fitted the model to the data by optimizing parameter k_c, compare figure S6. Based on this analysis, we concluded that the introduction of C as an intermediate species with two additional reaction rates renders the model non-identifiable. Furthermore, reaction rate k_b is in all cases about 25 to 26 fold larger than k_c, thus making reaction (1) above the limiting reaction for the formation of replication complexes. Since furthermore $k_a << k_c << k_b$, complexes C can be assumed to be almost constant at a low number, thus warranting an approximation using the ternary reaction $T_c + E_{cyt} + HF \rightarrow R_{Ip} + R_{ibo}$.

7. Stochastic effects in the viral life cycle

Since several of the species in our model attain low values, we assessed whether stochastic effects play an important role and possibly influence the model dynamics. For this purpose, we carried out stochastic simulations using the calibrated model and the implicit tau stochastic solver. Computations were carried out in Matlab, using the SimBiology toolbox. Supplementary figure S7 shows ten stochastic runs for high- and low permissive cells, indicating a very similar behavior to the deterministic differential equation model.

References

1. Huang da W, Sherman BT, Lempicki RA (2009) Systematic and integrative analysis of large gene lists using DAVID bioinformatics resources. Nature protocols 4: 44-57.
2. Huang da W, Sherman BT, Lempicki RA (2009) Bioinformatics enrichment tools: paths toward the comprehensive functional analysis of large gene lists. Nucleic acids research 37: 1-13.
3. Dahari H, Ribeiro RM, Rice CM, Perelson AS (2007) Mathematical modeling of subgenomic hepatitis C virus replication in Huh-7 cells. J Virol 81: 750-760.
4. Oh JW, Ito T, Lai MM (1999) A recombinant hepatitis C virus RNA-dependent RNA polymerase capable of copying the full-length viral RNA. J Virol 73: 7694-7702.
5. Ma H, Leveque V, De Witte A, Li W, Hendricks T, et al. (2005) Inhibition of native hepatitis C virus replicase by nucleotide and non-nucleoside inhibitors. Virology 332: 8-15.
6. Lohmann V, Korner F, Koch J, Herian U, Theilmann L, et al. (1999) Replication of subgenomic hepatitis C virus RNAs in a hepatoma cell line. Science 285: 110-113.
7. Thompson JF, Hayes LS, Lloyd DB (1991) Modulation of firefly luciferase stability and impact on studies of gene regulation. Gene 103: 171-177.
8. Leclerc GM, Boockfor FR, Faught WJ, Frawley LS (2000) Development of a destabilized firefly luciferase enzyme for measurement of gene expression. BioTechniques 29: 590-591, 594-596, 598 passim.
9. Pause A, Kukolj G, Bailey M, Brault M, Do F, et al. (2003) An NS3 serine protease inhibitor abrogates replication of subgenomic hepatitis C virus RNA. J Biol Chem 278: 20374-20380.
10. Pietschmann T, Lohmann V, Rutter G, Kurpanek K, Bartenschlager R (2001) Characterization of cell lines carrying self-replicating hepatitis C virus RNAs. J Virol 75: 1252-1264.
11. Wang C, Pflugheber J, Sumpter R, Jr., Sodora DL, Hui D, et al. (2003) Alpha interferon induces distinct translational control programs to suppress hepatitis C virus RNA replication. J Virol 77: 3898-3912.
12. Quinkert D, Bartenschlager R, Lohmann V (2005) Quantitative analysis of the hepatitis C virus replication complex. Journal of virology 79: 13594-13605.
13. Bock HG (1981) Numerical Treatment of inverse problems in chemical reaction kinetics. In: Ebert KH, Deuflhard P, Jäger W, editors. Modelling of Chemical Reaction Systems. Berlin, Heidelberg, New York: Springer. pp. 102-125.
14. Bock HG (1987) Randwertproblemmethoden zur Parameteridentifizierung in Systemen nichtlinearer Differentialgleichungen. Bonner Mathematische Schriften 183. Bonn.
15. Bock HG, Kostina EA, Schlöder JP (2007) Numerical Methods for Parameter Estimation in Nonlinear Differential Algebraic Equations. GAMM Mitteilungen 30(2): 376-408.
16. Baake E, Schlöder J (1992) Modelling the fast fluorescence rise of photosynthesis. Bull Math Biol 54: 999-1021.
17. Lenz SM, Bock HG, Schlöder JP, Kostina EA, Gienger G, et al. (2010) Multiple Shooting Method for Initial Satellite Orbit Determination. AIAA Journal on Guidance, Control and Dynamics 33: 1334-1346.
18. Bock HG, Kostina EA, Schlöder JP (2000) On the Role of Natural Level Functions to Achieve Global Convergence for Damped Newton Methods. In: Powell MJD, Scholtes S, editors. System Modelling and Optimization Methods, Theory and Applications. pp. 51-74.
19. Bader G, Deuflhard P (1983) A Semi-Implicit Mid-Point Rule for Stiff Systems of Ordinary Differential Equations. Numerische Mathematik 41: 373-398.
20. Rodriguez-Fernandez M, Banga JR (2010) SensSB: a software toolbox for the development and sensitivity analysis of systems biology models. Bioinformatics 26: 1675-1676.
21. Marino S, Hogue IB, Ray CJ, Kirschner DE (2008) A methodology for performing global uncertainty and sensitivity analysis in systems biology. J Theor Biol 254: 178-196.
22. Saltelli A, Bolado R (1998) An alternative way to compute Fourier amplitude sensitivity test (FAST). Comput Stat Data Anal 26 (4), 445–460.: 445–460.
